# Supplementary material for: An ancient bacterial zinc acquisition system identified from a cyanobacterial exoproteome
Source: PLoS Biol. 2024 Mar 11;22(3):e3002546. doi: 10.1371/journal.pbio.3002546 (PMC10957091; doi:10.1371/journal.pbio.3002546)
Supplement: S1 Fig — Scatter plots showing the correlation between the normalized protein intensities (log2 transformed) of the 2 biological replicates of Anabaena sp. PCC 7120 (WT) (A) and Δzur strain (B). Pearson’s correlation coefficient (R2) is shown. The data underlying this figure can be found in S1 Data. (PPTX) [file pbio.3002546.s001.pptx]

## Slide 1
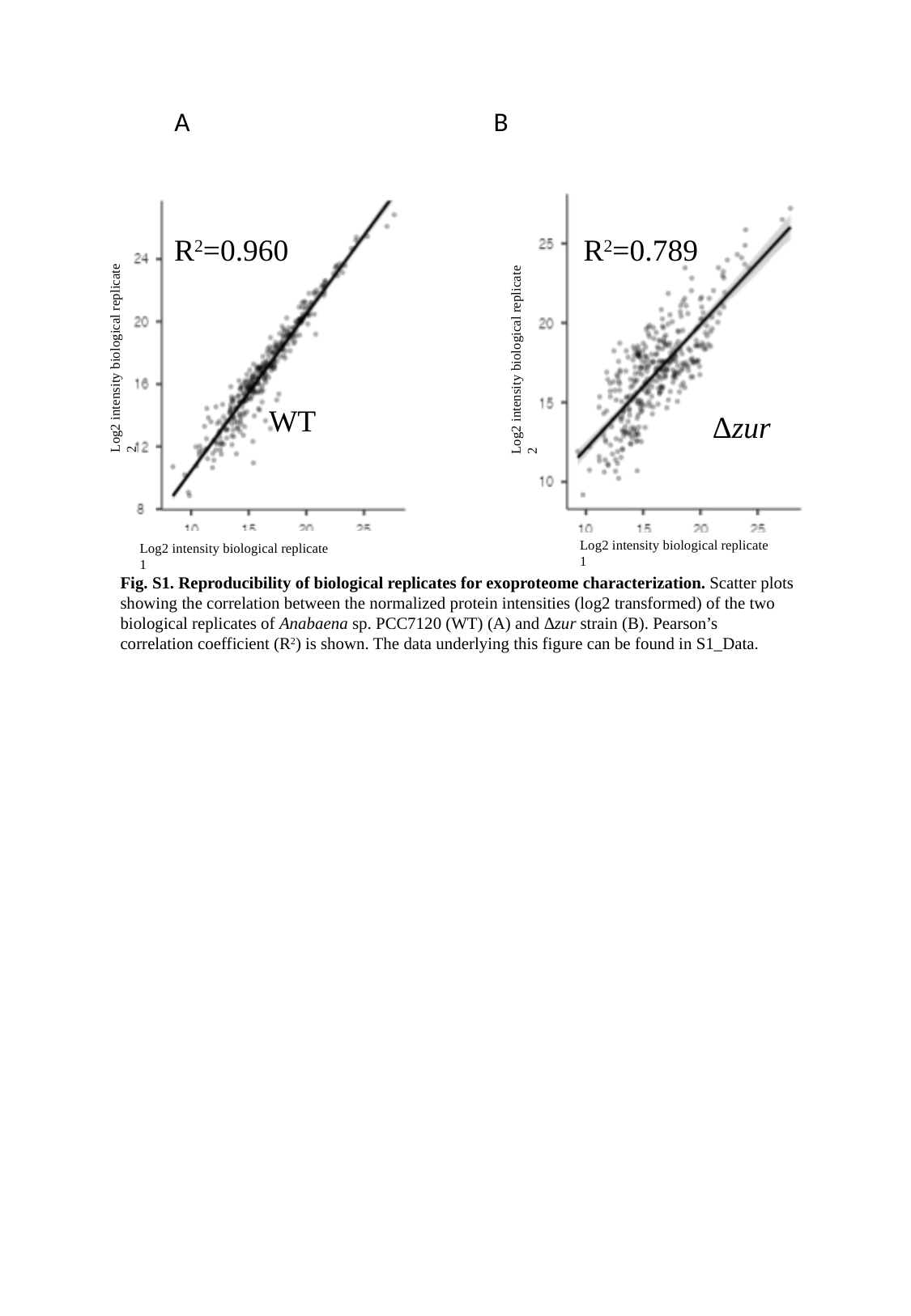

A
B
R2=0.960
R2=0.789
Log2 intensity biological replicate 2
Log2 intensity biological replicate 2
WT
Δzur
Log2 intensity biological replicate 1
Log2 intensity biological replicate 1
Fig. S1. Reproducibility of biological replicates for exoproteome characterization. Scatter plots showing the correlation between the normalized protein intensities (log2 transformed) of the two biological replicates of Anabaena sp. PCC7120 (WT) (A) and Δzur strain (B). Pearson’s correlation coefficient (R2) is shown. The data underlying this figure can be found in S1_Data.
