# Supplementary material for: An ancient bacterial zinc acquisition system identified from a cyanobacterial exoproteome
Source: PLoS Biol. 2024 Mar 11;22(3):e3002546. doi: 10.1371/journal.pbio.3002546 (PMC10957091; doi:10.1371/journal.pbio.3002546)
Supplement: S2 Fig — (A) Sequence of All3515/ZepA showing the N-terminal signal peptide and the C-terminal PEP-CTERM domain. Histidine residues predicted to coordinate a zinc atom are depicted in pink color, histidine residues of the N-terminus of the mature protein are shown in orange color and acidic residues in this region are in green. (B) Comparison of the C-terminal All3515 sequence to the archetypal PEP-CTERM domain described by Haft and colleagues. (C) A scheme indicating the steps followed for the construction of the all3515 mutants is shown. Segregation analysis of the mutants is shown at the bottom of the figure. Vertical dashed lines on the gel image are included solely to indicate that irrelevant parts of the gels are not shown. (D) Western blot of cell fractions of E. coli Lemo21(DE3) pET28b:ZepA_Ana and the Δzur mutant of Anabaena. Lanes 1–5 were loaded with fractions from E. coli Lemo21(DE3) pET28b:ZepA_Ana. Lane 1 was loaded with a cell extract (CE) corresponding to 50 μl of culture, lanes 2 and 3 were loaded with cell extract (CE) or spheroplast extract (Sphe) corresponding to 40 μl of culture, lane 4 was loaded with periplasmic fraction (Peripl) corresponding to 500 μl of culture, lane 5 was loaded with extracellular material (EM) corresponding to 1.25 ml of culture, lane 6 was loaded with extracellular material corresponding to 6 ml of culture of the Δzur mutant of Anabaena. The gel of the top panel was subjected to western blot with a specific antibody against ZepA. Unspecific bands are labeled as “u.” Bands reacting specifically to the anti-ZepA antibody are labeled as “a,” “b,” or “c” according to their size. A vertical dashed line is depicted to indicate that parts at each side of the line were exposed differently. The bottom panel is an identical gel stained with Coomassie. (PPTX) [file pbio.3002546.s002.pptx]

## Slide 1
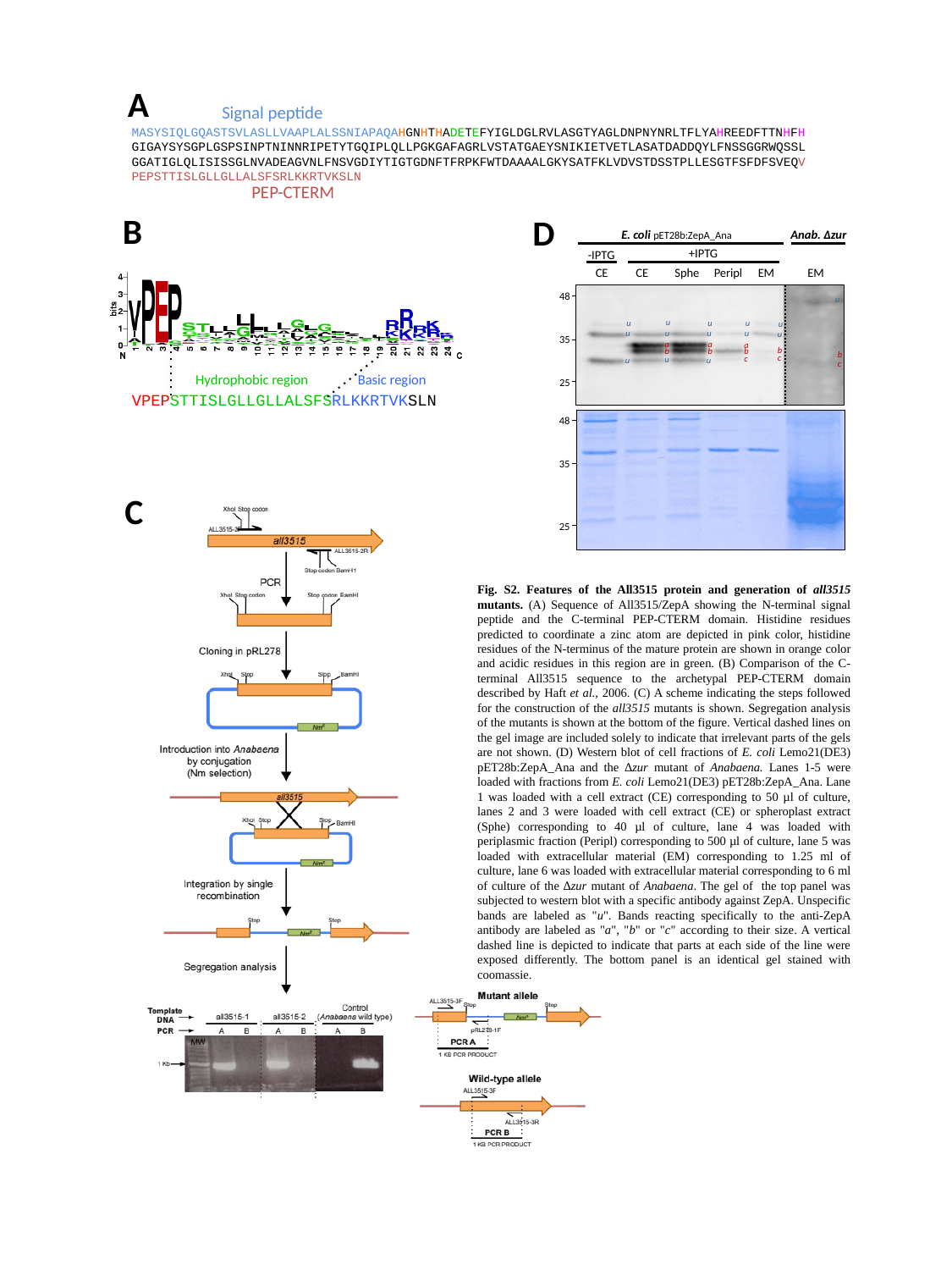

A
Signal peptide
MASYSIQLGQASTSVLASLLVAAPLALSSNIAPAQAHGNHTHADETEFYIGLDGLRVLASGTYAGLDNPNYNRLTFLYAHREEDFTTNHFHGIGAYSYSGPLGSPSINPTNINNRIPETYTGQIPLQLLPGKGAFAGRLVSTATGAEYSNIKIETVETLASATDADDQYLFNSSGGRWQSSLGGATIGLQLISISSGLNVADEAGVNLFNSVGDIYTIGTGDNFTFRPKFWTDAAAALGKYSATFKLVDVSTDSSTPLLESGTFSFDFSVEQVPEPSTTISLGLLGLLALSFSRLKKRTVKSLN
PEP-CTERM
B
D
E. coli pET28b:ZepA_Ana
Anab. ∆zur
+IPTG
-IPTG
CE
CE
Sphe
Peripl
EM
EM
Hydrophobic region
Basic region
VPEPSTTISLGLLGLLALSFSRLKKRTVKSLN
48
u
u
u
u
u
u
u
u
u
u
u
35
a
a
a
b
b
b
b
b
c
c
u
u
u
c
25
48
35
C
25
Fig. S2. Features of the All3515 protein and generation of all3515 mutants. (A) Sequence of All3515/ZepA showing the N-terminal signal peptide and the C-terminal PEP-CTERM domain. Histidine residues predicted to coordinate a zinc atom are depicted in pink color, histidine residues of the N-terminus of the mature protein are shown in orange color and acidic residues in this region are in green. (B) Comparison of the C-terminal All3515 sequence to the archetypal PEP-CTERM domain described by Haft et al., 2006. (C) A scheme indicating the steps followed for the construction of the all3515 mutants is shown. Segregation analysis of the mutants is shown at the bottom of the figure. Vertical dashed lines on the gel image are included solely to indicate that irrelevant parts of the gels are not shown. (D) Western blot of cell fractions of E. coli Lemo21(DE3) pET28b:ZepA_Ana and the ∆zur mutant of Anabaena. Lanes 1-5 were loaded with fractions from E. coli Lemo21(DE3) pET28b:ZepA_Ana. Lane 1 was loaded with a cell extract (CE) corresponding to 50 µl of culture, lanes 2 and 3 were loaded with cell extract (CE) or spheroplast extract (Sphe) corresponding to 40 µl of culture, lane 4 was loaded with periplasmic fraction (Peripl) corresponding to 500 µl of culture, lane 5 was loaded with extracellular material (EM) corresponding to 1.25 ml of culture, lane 6 was loaded with extracellular material corresponding to 6 ml of culture of the ∆zur mutant of Anabaena. The gel of the top panel was subjected to western blot with a specific antibody against ZepA. Unspecific bands are labeled as "u". Bands reacting specifically to the anti-ZepA antibody are labeled as "a", "b" or "c" according to their size. A vertical dashed line is depicted to indicate that parts at each side of the line were exposed differently. The bottom panel is an identical gel stained with coomassie.
