# Supplementary material for: An ancient bacterial zinc acquisition system identified from a cyanobacterial exoproteome
Source: PLoS Biol. 2024 Mar 11;22(3):e3002546. doi: 10.1371/journal.pbio.3002546 (PMC10957091; doi:10.1371/journal.pbio.3002546)
Supplement: S3 Fig — The picture shows a theoretical model based on results presented in this manuscript of zinc acquisition mediated by ZepA in Anabaena. Zinc atoms are depicted as yellow dots. OM stands for outer membrane and PM for plasma membrane. Cylinders on the outer membrane represent Zur-regulated TBDTs All3242 and All4028-4029. Dashed arrows indicate steps that will need to be further investigated. (PPTX) [file pbio.3002546.s003.pptx]

## Slide 1
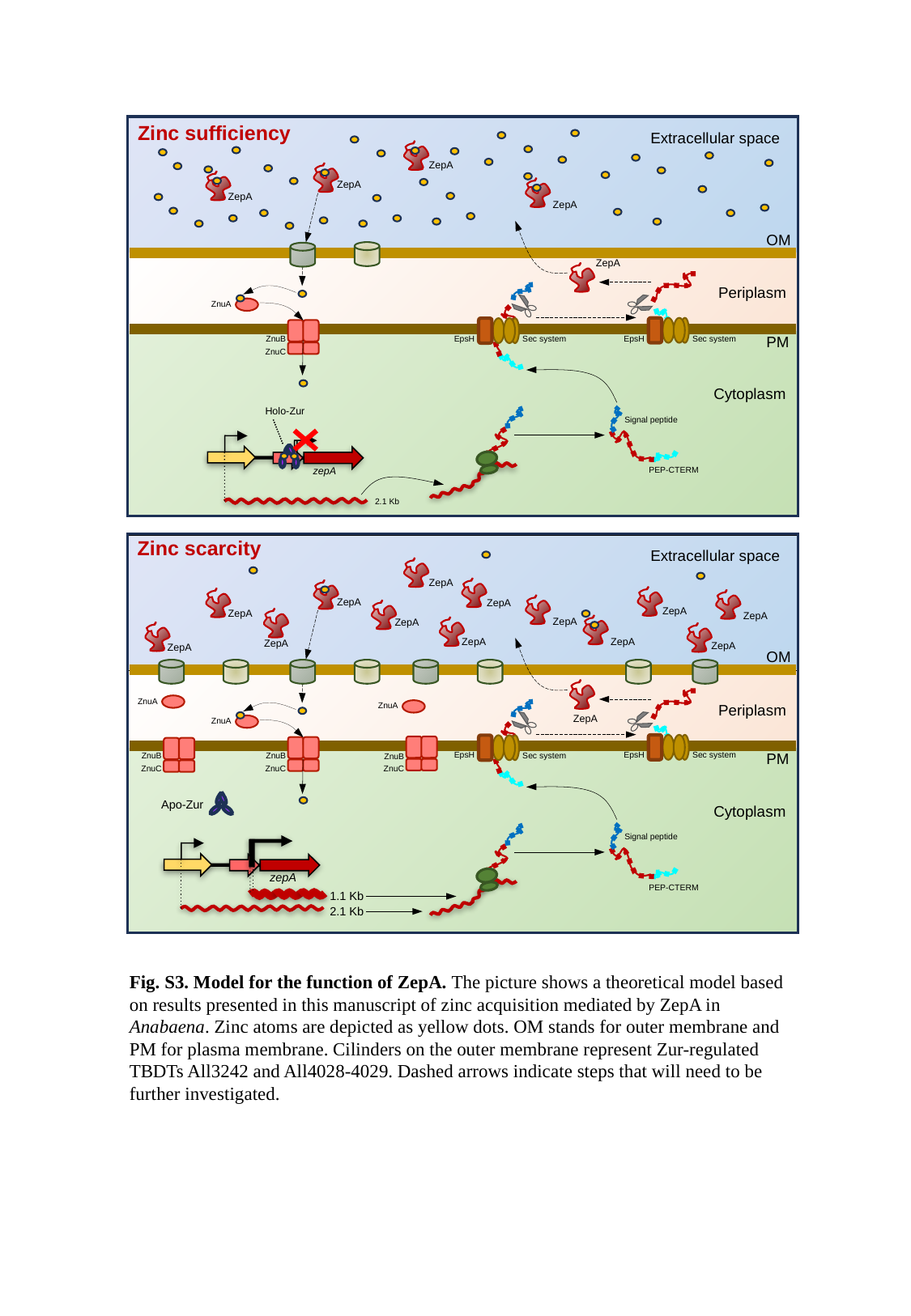

Zinc sufficiency
Extracellular space
ZepA
ZepA
ZepA
ZepA
OM
ZepA
Periplasm
ZnuA
EpsH
Sec system
EpsH
Sec system
PM
ZnuB
ZnuC
Cytoplasm
Holo-Zur
zepA
2.1 Kb
Signal peptide
PEP-CTERM
Zinc scarcity
Extracellular space
ZepA
ZepA
ZepA
ZepA
ZepA
ZepA
ZepA
ZepA
ZepA
ZepA
ZepA
ZepA
ZepA
OM
ZnuA
Periplasm
ZnuA
ZepA
ZnuA
EpsH
Sec system
EpsH
Sec system
PM
ZnuB
ZnuB
ZnuB
ZnuC
ZnuC
ZnuC
Apo-Zur
Cytoplasm
Signal peptide
zepA
PEP-CTERM
1.1 Kb
2.1 Kb
Fig. S3. Model for the function of ZepA. The picture shows a theoretical model based on results presented in this manuscript of zinc acquisition mediated by ZepA in Anabaena. Zinc atoms are depicted as yellow dots. OM stands for outer membrane and PM for plasma membrane. Cilinders on the outer membrane represent Zur-regulated TBDTs All3242 and All4028-4029. Dashed arrows indicate steps that will need to be further investigated.
