# Supplementary material for: An ancient bacterial zinc acquisition system identified from a cyanobacterial exoproteome
Source: PLoS Biol. 2024 Mar 11;22(3):e3002546. doi: 10.1371/journal.pbio.3002546 (PMC10957091; doi:10.1371/journal.pbio.3002546)
Supplement: S4 Fig — (A) Coverage and identity analysis of the multiple sequence alignment provided to Alphafold2 for structure prediction. Two juxtaposed plots are shown in this panel. One plot shows the coverage and identity of sequence homologs represented as complete or interrupted horizontal lines ordered from less (bottom) to more (top) conserved with respect to the mature sequence of ZepA of Anabaena (the query). Each line is depicted according to the color scale of the sequence identity shown on the right side. Gaps in the alignment are shown as empty spaces. The second plot in this left panel corresponds to the black line, which represents the number of sequences (left vertical axis) in which a given position of the query protein is conserved. The right panel is similar but uses the ZepA protein from Lacipirellula limnantheis (PVC superphylum) as the query protein. In this case, homologous sequences to the query protein were selected by Alphafold2 (mmseqs2_uniref_env setting). (B) Predicted lDDT per position on a scale from 0–100 of 5 AlphaFold2 models colored by rank. The average pLDDT of the most confident prediction (rank1) is 93.5 (for Anabaena) and 95.2 (for Lacipirellula). Model confidence varies significantly along the chain, but most positions are modeled with high accuracy (pLDDT > 90) and can serve to characterize binding sites. In the 5 models, accuracy decreased between 30 and 70 at the 10 N-terminal amino acids (pLDDT < 50) predicting a disordered region (unstructured under physiological conditions). The yellow inset represents the output of the MIB2 metal binding prediction server showing metal binding scores for each amino acid (derived from sequence and structure conservation measures) as a yellow line. Scores higher than 2 predict a metal-binding residue (red circle). Vertical gray bands were depicted to show that predicted metal binding sites are located at regions where the model confidence is lower, which also coincides with regions of low sequence conservat [file pbio.3002546.s004.pptx]

## Slide 1
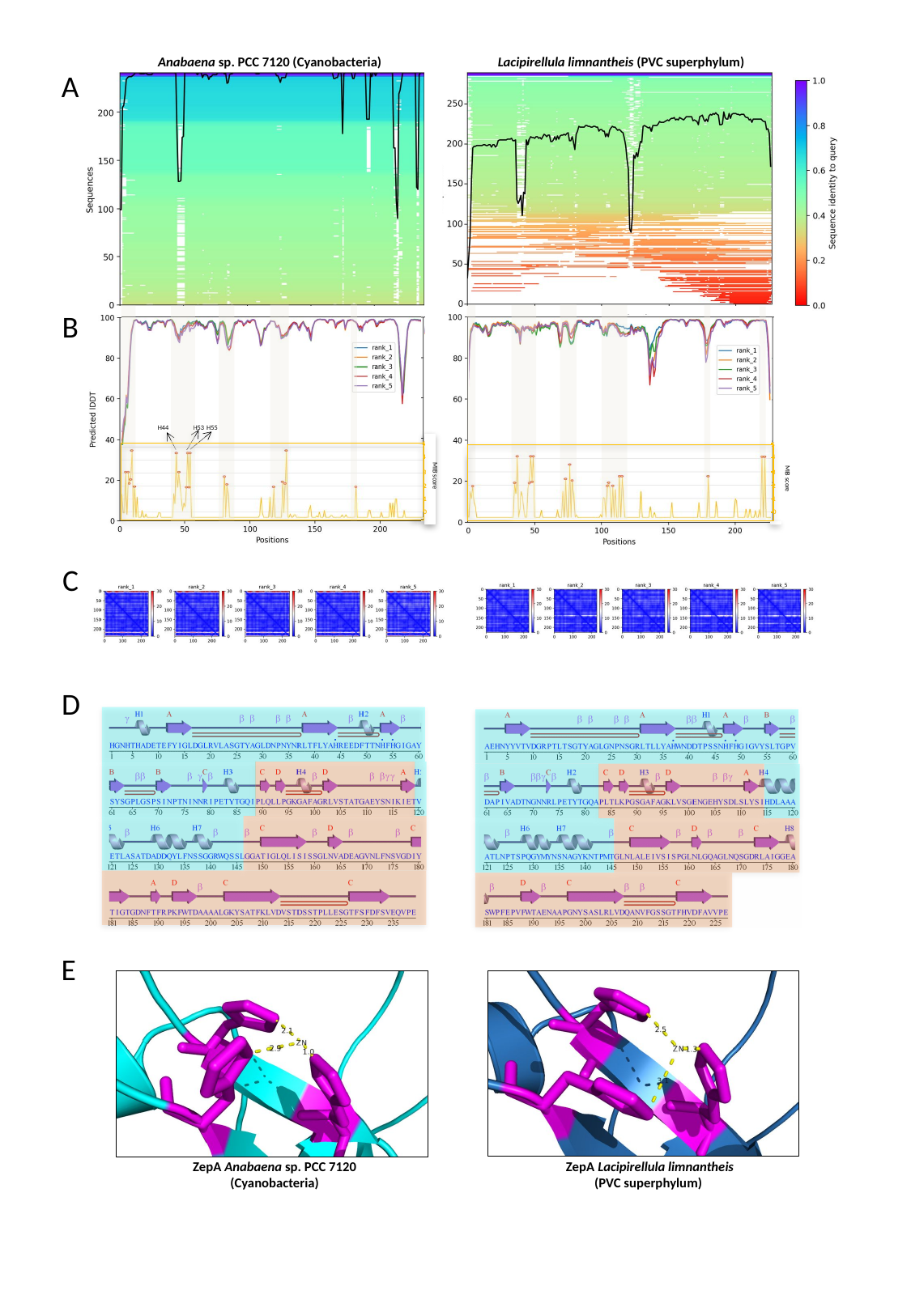

Anabaena sp. PCC 7120 (Cyanobacteria)
Lacipirellula limnantheis (PVC superphylum)
A
B
H53
H55
H44
5
4
3
2
1
0
5
4
3
2
1
0
MIB score
MIB score
C
D
E
ZepA Anabaena sp. PCC 7120
(Cyanobacteria)
ZepA Lacipirellula limnantheis
(PVC superphylum)

## Slide 2
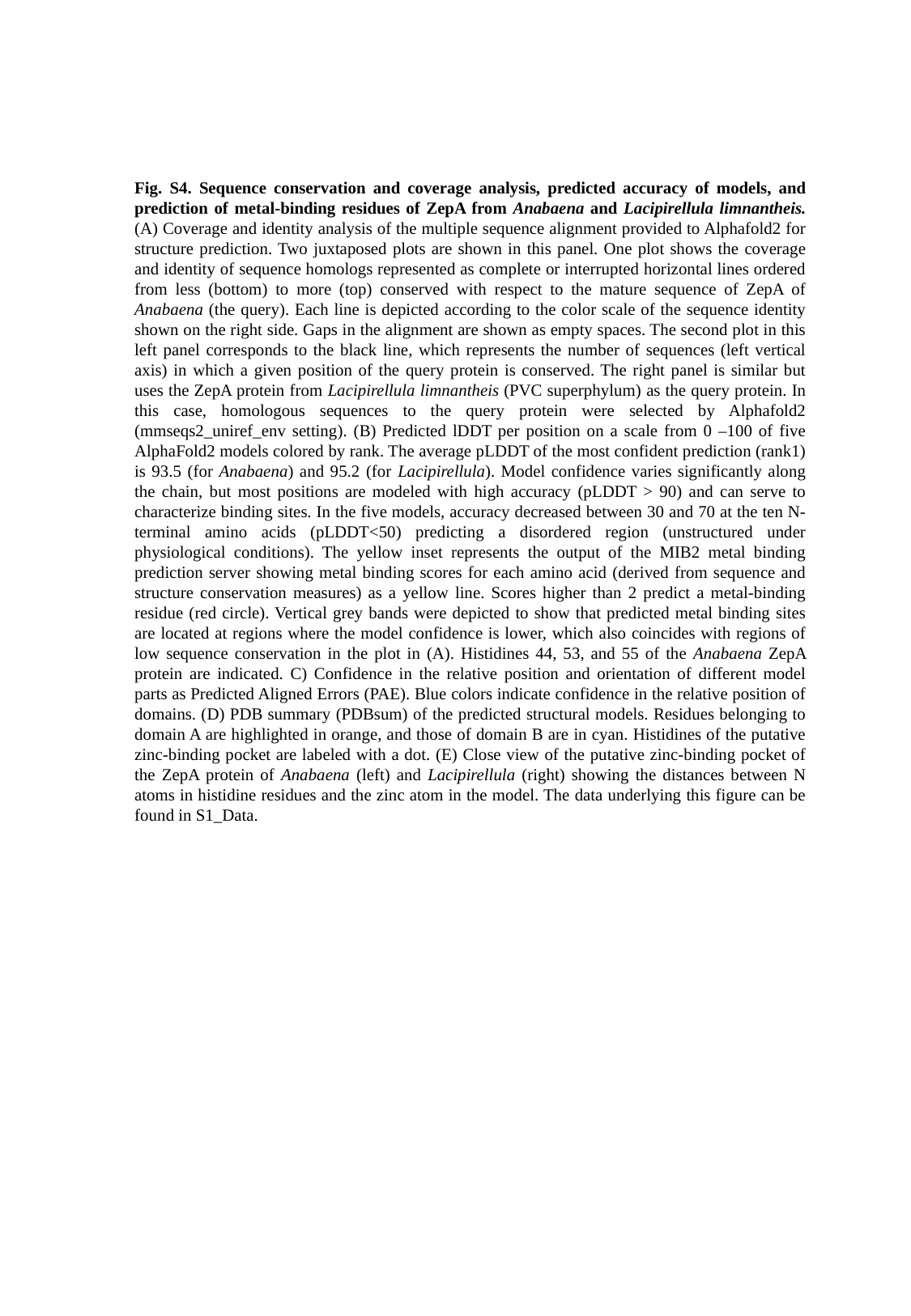

Fig. S4. Sequence conservation and coverage analysis, predicted accuracy of models, and prediction of metal-binding residues of ZepA from Anabaena and Lacipirellula limnantheis. (A) Coverage and identity analysis of the multiple sequence alignment provided to Alphafold2 for structure prediction. Two juxtaposed plots are shown in this panel. One plot shows the coverage and identity of sequence homologs represented as complete or interrupted horizontal lines ordered from less (bottom) to more (top) conserved with respect to the mature sequence of ZepA of Anabaena (the query). Each line is depicted according to the color scale of the sequence identity shown on the right side. Gaps in the alignment are shown as empty spaces. The second plot in this left panel corresponds to the black line, which represents the number of sequences (left vertical axis) in which a given position of the query protein is conserved. The right panel is similar but uses the ZepA protein from Lacipirellula limnantheis (PVC superphylum) as the query protein. In this case, homologous sequences to the query protein were selected by Alphafold2 (mmseqs2_uniref_env setting). (B) Predicted lDDT per position on a scale from 0 –100 of five AlphaFold2 models colored by rank. The average pLDDT of the most confident prediction (rank1) is 93.5 (for Anabaena) and 95.2 (for Lacipirellula). Model confidence varies significantly along the chain, but most positions are modeled with high accuracy (pLDDT > 90) and can serve to characterize binding sites. In the five models, accuracy decreased between 30 and 70 at the ten N-terminal amino acids (pLDDT<50) predicting a disordered region (unstructured under physiological conditions). The yellow inset represents the output of the MIB2 metal binding prediction server showing metal binding scores for each amino acid (derived from sequence and structure conservation measures) as a yellow line. Scores higher than 2 predict a metal-binding residue (red circle). Vertical grey bands were depicted to show that predicted metal binding sites are located at regions where the model confidence is lower, which also coincides with regions of low sequence conservation in the plot in (A). Histidines 44, 53, and 55 of the Anabaena ZepA protein are indicated. C) Confidence in the relative position and orientation of different model parts as Predicted Aligned Errors (PAE). Blue colors indicate confidence in the relative position of domains. (D) PDB summary (PDBsum) of the predicted structural models. Residues belonging to domain A are highlighted in orange, and those of domain B are in cyan. Histidines of the putative zinc-binding pocket are labeled with a dot. (E) Close view of the putative zinc-binding pocket of the ZepA protein of Anabaena (left) and Lacipirellula (right) showing the distances between N atoms in histidine residues and the zinc atom in the model. The data underlying this figure can be found in S1_Data.
