# Supplementary material for: An ancient bacterial zinc acquisition system identified from a cyanobacterial exoproteome
Source: PLoS Biol. 2024 Mar 11;22(3):e3002546. doi: 10.1371/journal.pbio.3002546 (PMC10957091; doi:10.1371/journal.pbio.3002546)
Supplement: S5 Fig — (A) Structural models for the ZepA proteins from the indicated organisms were built using AlphaFold2 and are shown. (B) Structural models were aligned with PyMOL v 1.7.6.3 and are shown in different orientations. (C) Close view of the Anabaena ZepA model showing residues putatively involved in zinc coordination. (PPTX) [file pbio.3002546.s005.pptx]

## Slide 1
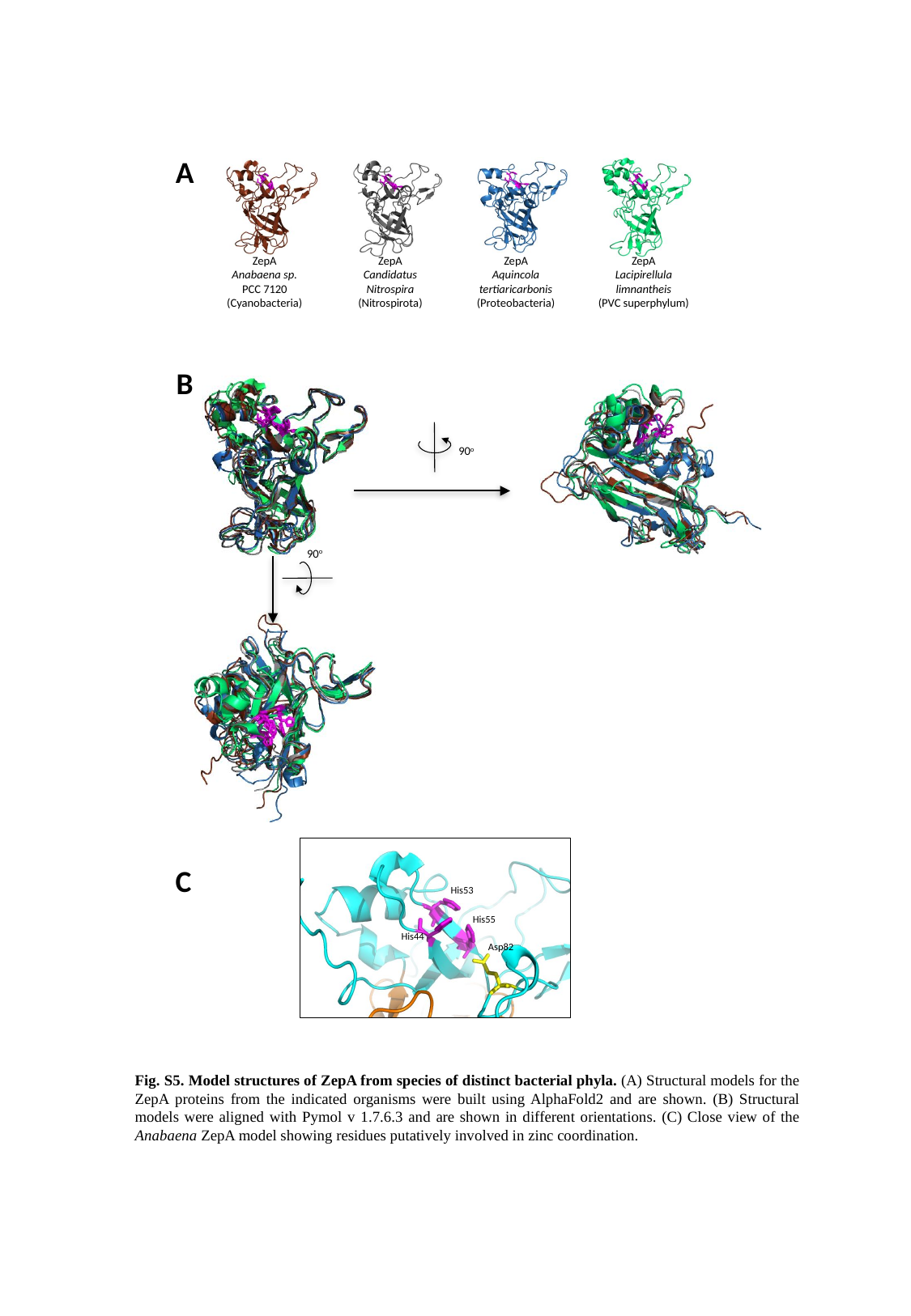

A
ZepA
Anabaena sp. PCC 7120
(Cyanobacteria)
ZepA
Candidatus Nitrospira
(Nitrospirota)
ZepA
Aquincola tertiaricarbonis
(Proteobacteria)
ZepA
Lacipirellula
limnantheis
(PVC superphylum)
B
90o
90o
C
His53
His55
His44
Asp82
Fig. S5. Model structures of ZepA from species of distinct bacterial phyla. (A) Structural models for the ZepA proteins from the indicated organisms were built using AlphaFold2 and are shown. (B) Structural models were aligned with Pymol v 1.7.6.3 and are shown in different orientations. (C) Close view of the Anabaena ZepA model showing residues putatively involved in zinc coordination.
