# Supplementary material for: An ancient bacterial zinc acquisition system identified from a cyanobacterial exoproteome
Source: PLoS Biol. 2024 Mar 11;22(3):e3002546. doi: 10.1371/journal.pbio.3002546 (PMC10957091; doi:10.1371/journal.pbio.3002546)
Supplement: S6 Fig — (A) Plots correspond to the output of the MIB2 metal binding prediction server. The y axis indicates the MIB2 score and the x axis indicates the amino acid positions. The species and the phylum of each ZepA homolog is indicated. Histidines predicted to form the zinc-binding pocket are enclosed in a frame and indicated with a purple bar. Notice that in Gloeothece verrucosa, only 2 of the 3 histidine residues are conserved. The consensus sequence of the putative zinc-binding pocket is shown at the top with histidines residues in purple. The data underlying this figure can be found in S1 Data. (B) Pictures show a close view of the zinc-binding pocket of each ZepA homolog. The structure of each protein was modeled with AlphaFold2. Putative zinc-binding histidine residues are depicted in purple color. (PPTX) [file pbio.3002546.s006.pptx]

## Slide 1
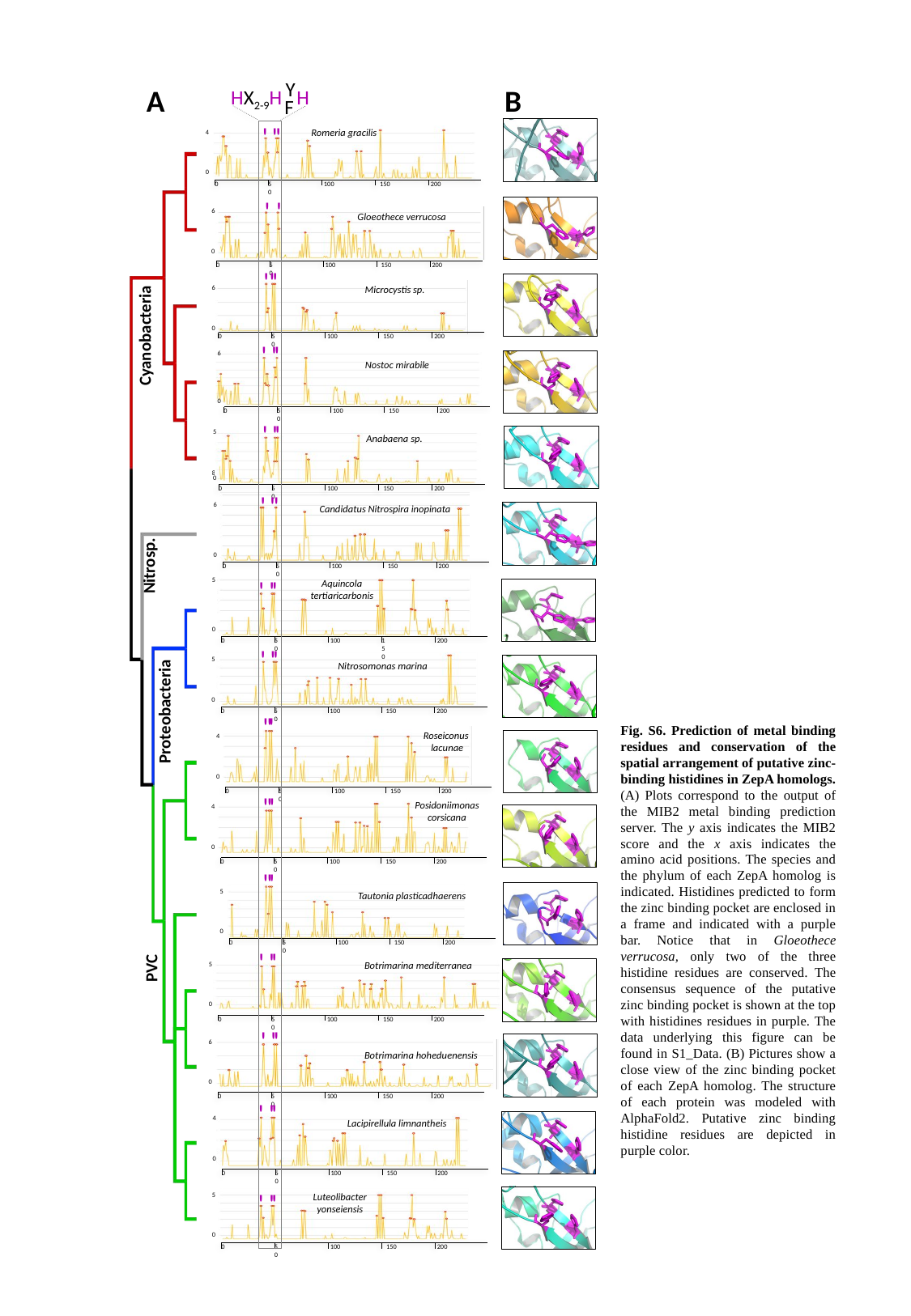

Y
F
HX2-9H H
A
B
'
'
'
Romeria gracilis
4
0
0
50
100
150
200
'
'
6
Gloeothece verrucosa
0
0
50
100
150
200
'
'
'
6
Microcystis sp.
0
0
50
100
150
200
Cyanobacteria
'
'
'
6
0
0
50
100
150
200
Nostoc mirabile
'
'
'
5
8
0
0
50
100
150
200
Anabaena sp.
'
'
'
6
0
0
50
100
150
200
Candidatus Nitrospira inopinata
Nitrosp.
'
'
'
5
0
0
50
100
150
200
Aquincola
tertiaricarbonis
'
'
'
5
0
0
50
100
150
200
Nitrosomonas marina
Proteobacteria
'
'
'
Roseiconus
lacunae
4
0
0
50
100
150
200
'
'
'
Posidoniimonas
corsicana
4
0
0
50
100
150
200
'
'
'
5
0
0
50
100
150
200
Tautonia plasticadhaerens
'
'
'
PVC
Botrimarina mediterranea
5
0
0
50
100
150
200
'
'
'
6
0
0
50
100
150
200
Botrimarina hoheduenensis
'
'
'
4
0
0
50
100
150
200
Lacipirellula limnantheis
'
'
'
5
0
0
50
100
150
200
Luteolibacter
yonseiensis
Fig. S6. Prediction of metal binding residues and conservation of the spatial arrangement of putative zinc-binding histidines in ZepA homologs. (A) Plots correspond to the output of the MIB2 metal binding prediction server. The y axis indicates the MIB2 score and the x axis indicates the amino acid positions. The species and the phylum of each ZepA homolog is indicated. Histidines predicted to form the zinc binding pocket are enclosed in a frame and indicated with a purple bar. Notice that in Gloeothece verrucosa, only two of the three histidine residues are conserved. The consensus sequence of the putative zinc binding pocket is shown at the top with histidines residues in purple. The data underlying this figure can be found in S1_Data. (B) Pictures show a close view of the zinc binding pocket of each ZepA homolog. The structure of each protein was modeled with AlphaFold2. Putative zinc binding histidine residues are depicted in purple color.
