# Supplementary material for: An ancient bacterial zinc acquisition system identified from a cyanobacterial exoproteome
Source: PLoS Biol. 2024 Mar 11;22(3):e3002546. doi: 10.1371/journal.pbio.3002546 (PMC10957091; doi:10.1371/journal.pbio.3002546)
Supplement: S7 Fig — The structure of these proteins predicted by AlphaFold2 was submitted to the Galaxysite server (https://openebench.bsc.es/tool/galaxysite). Molecules predicted to interact with these proteins are shown together with the localization of the docking site. We interpret that the planar lipid, would likely correspond to a hopanoid instead of a planar sterol as predicted by the Galaxysite server. Planar sterols are rare in bacteria but are highly similar to bacterial hopanoids. Notice that the 2 structures are viewed from distinct positions. (PPTX) [file pbio.3002546.s007.pptx]

## Slide 1
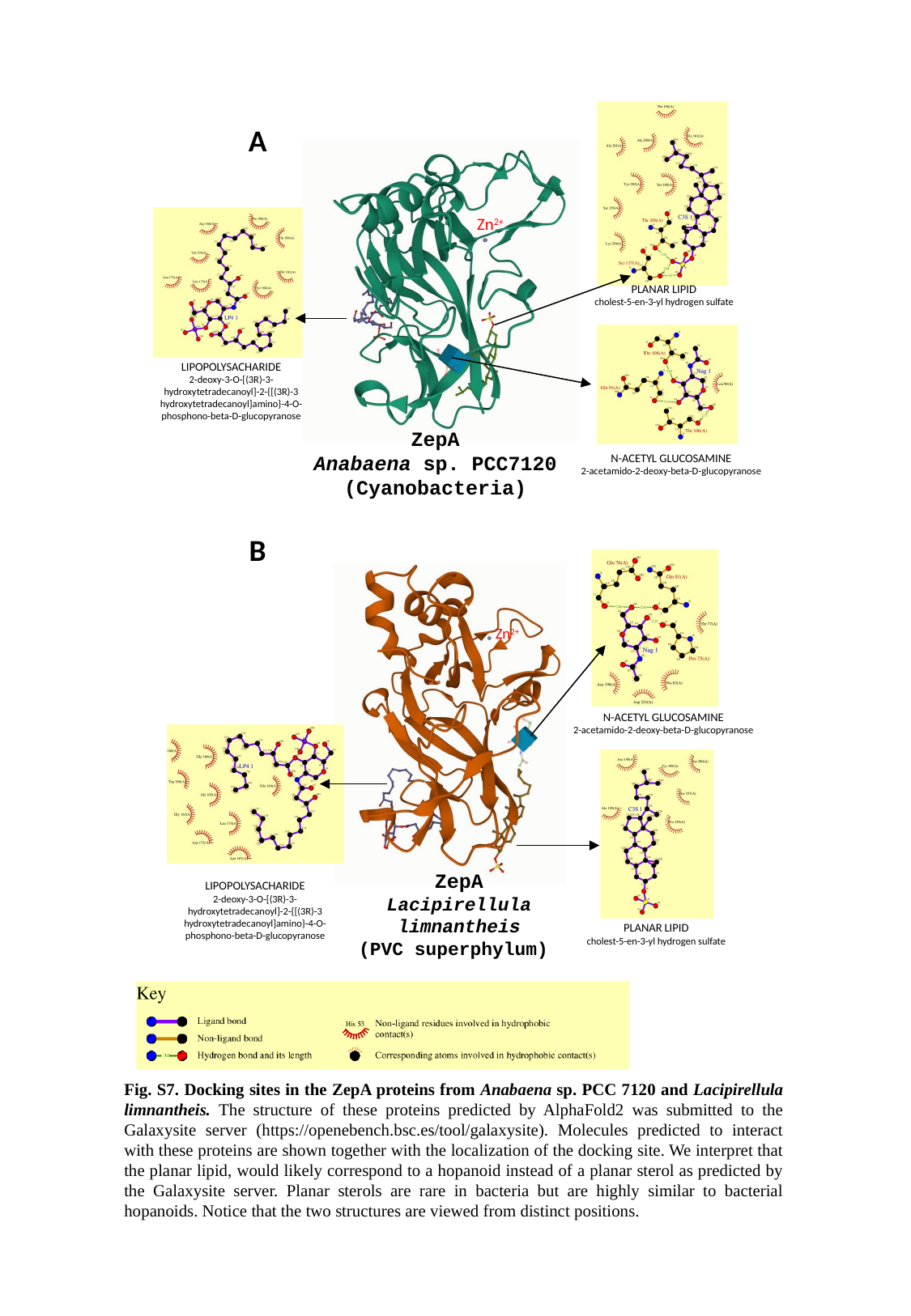

A
Zn2+
PLANAR LIPID
cholest-5-en-3-yl hydrogen sulfate
LIPOPOLYSACHARIDE
2-deoxy-3-O-[(3R)-3-hydroxytetradecanoyl]-2-{[(3R)-3 hydroxytetradecanoyl]amino}-4-O-phosphono-beta-D-glucopyranose
ZepA
Anabaena sp. PCC7120
(Cyanobacteria)
N-ACETYL GLUCOSAMINE
2-acetamido-2-deoxy-beta-D-glucopyranose
B
Zn2+
N-ACETYL GLUCOSAMINE
2-acetamido-2-deoxy-beta-D-glucopyranose
ZepA
Lacipirellula limnantheis
(PVC superphylum)
LIPOPOLYSACHARIDE
2-deoxy-3-O-[(3R)-3-hydroxytetradecanoyl]-2-{[(3R)-3 hydroxytetradecanoyl]amino}-4-O-phosphono-beta-D-glucopyranose
PLANAR LIPID
cholest-5-en-3-yl hydrogen sulfate
Fig. S7. Docking sites in the ZepA proteins from Anabaena sp. PCC 7120 and Lacipirellula limnantheis. The structure of these proteins predicted by AlphaFold2 was submitted to the Galaxysite server (https://openebench.bsc.es/tool/galaxysite). Molecules predicted to interact with these proteins are shown together with the localization of the docking site. We interpret that the planar lipid, would likely correspond to a hopanoid instead of a planar sterol as predicted by the Galaxysite server. Planar sterols are rare in bacteria but are highly similar to bacterial hopanoids. Notice that the two structures are viewed from distinct positions.
