# Supplementary material for: An ancient bacterial zinc acquisition system identified from a cyanobacterial exoproteome
Source: PLoS Biol. 2024 Mar 11;22(3):e3002546. doi: 10.1371/journal.pbio.3002546 (PMC10957091; doi:10.1371/journal.pbio.3002546)
Supplement: S8 Fig — Structural models obtained with AlphaFold2 for the ZepA protein of the indicated species were used to interrogate the MIB server or the GalaxySite server to determine putative interaction sites. Conserved sites are shown as solid boxed and absent sites as empty boxes. Zn, zinc; LP4, a lipopolisacharide (2-deoxy-3-O-[(3R)-3-hydroxytetradecanoyl]-2-{[(3R)-3-hydroxytetradecanoyl]amino}-4-O-phosphono-beta-D-glucopyranose); C3S, a planar sterol (cholest-5-en-3-yl hydrogen sulfate); NAG, N-acetyl glucosamine (2-acetamido-2-deoxy-beta-D-glucopyranose); MAN (alpha-D-mannopyranose); OLA (oleic acid). (PPTX) [file pbio.3002546.s008.pptx]

## Slide 1
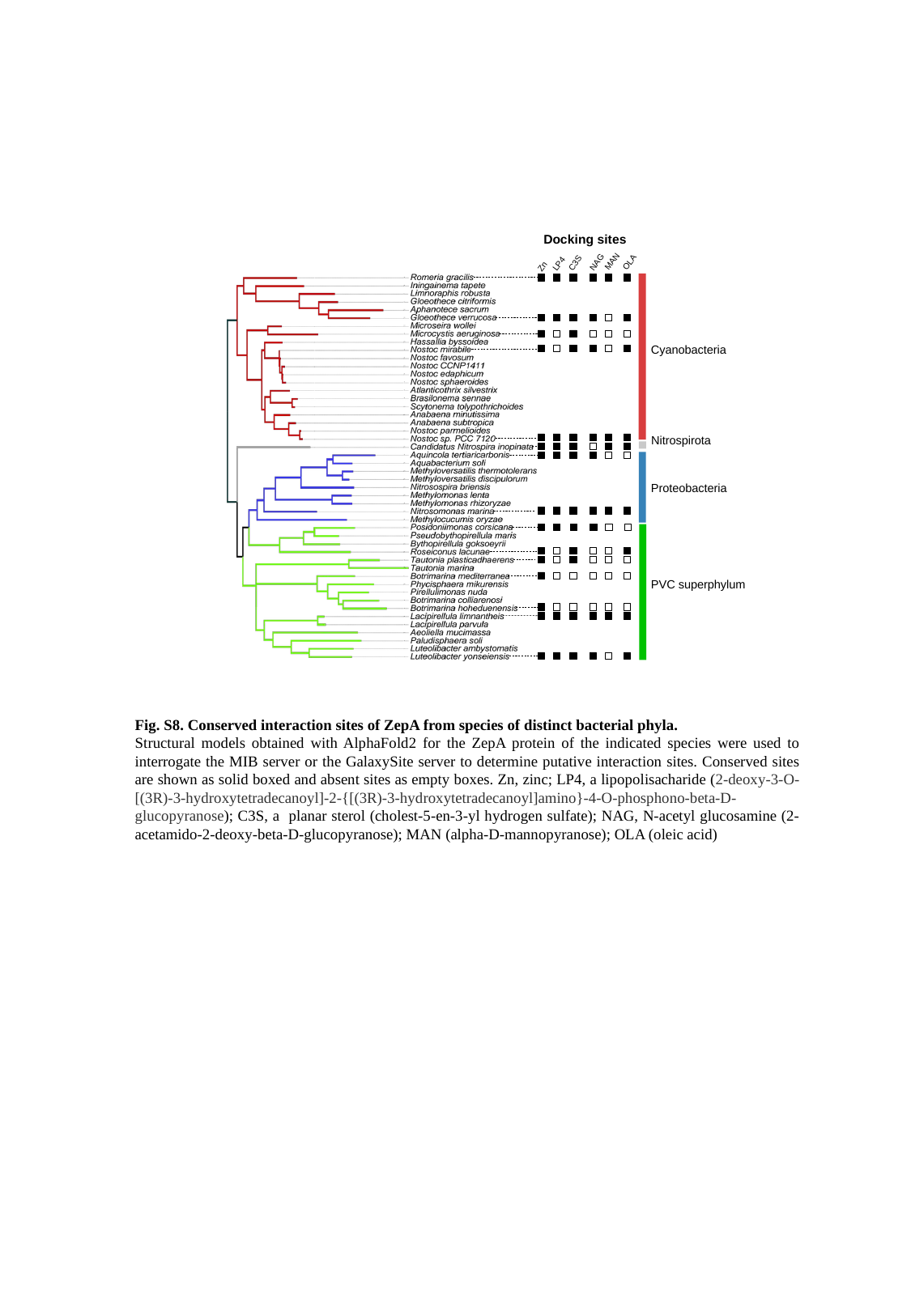

Docking sites
NAG
MAN
OLA
LP4
C3S
Zn
Cyanobacteria
Nitrospirota
Proteobacteria
PVC superphylum
Fig. S8. Conserved interaction sites of ZepA from species of distinct bacterial phyla.
Structural models obtained with AlphaFold2 for the ZepA protein of the indicated species were used to interrogate the MIB server or the GalaxySite server to determine putative interaction sites. Conserved sites are shown as solid boxed and absent sites as empty boxes. Zn, zinc; LP4, a lipopolisacharide (2-deoxy-3-O-[(3R)-3-hydroxytetradecanoyl]-2-{[(3R)-3-hydroxytetradecanoyl]amino}-4-O-phosphono-beta-D-glucopyranose); C3S, a planar sterol (cholest-5-en-3-yl hydrogen sulfate); NAG, N-acetyl glucosamine (2-acetamido-2-deoxy-beta-D-glucopyranose); MAN (alpha-D-mannopyranose); OLA (oleic acid)
