# Supplementary material for: An ancient bacterial zinc acquisition system identified from a cyanobacterial exoproteome
Source: PLoS Biol. 2024 Mar 11;22(3):e3002546. doi: 10.1371/journal.pbio.3002546 (PMC10957091; doi:10.1371/journal.pbio.3002546)
Supplement: S9 Fig — Species names are connected to their corresponding branch by solid or dashed gray lines. As shown, the position of phyla in both trees mirrored each other, which is indicative of rare horizontal transfer events between phyla. The data underlying this figure can be found in S1 Data. (PPTX) [file pbio.3002546.s009.pptx]

## Slide 1
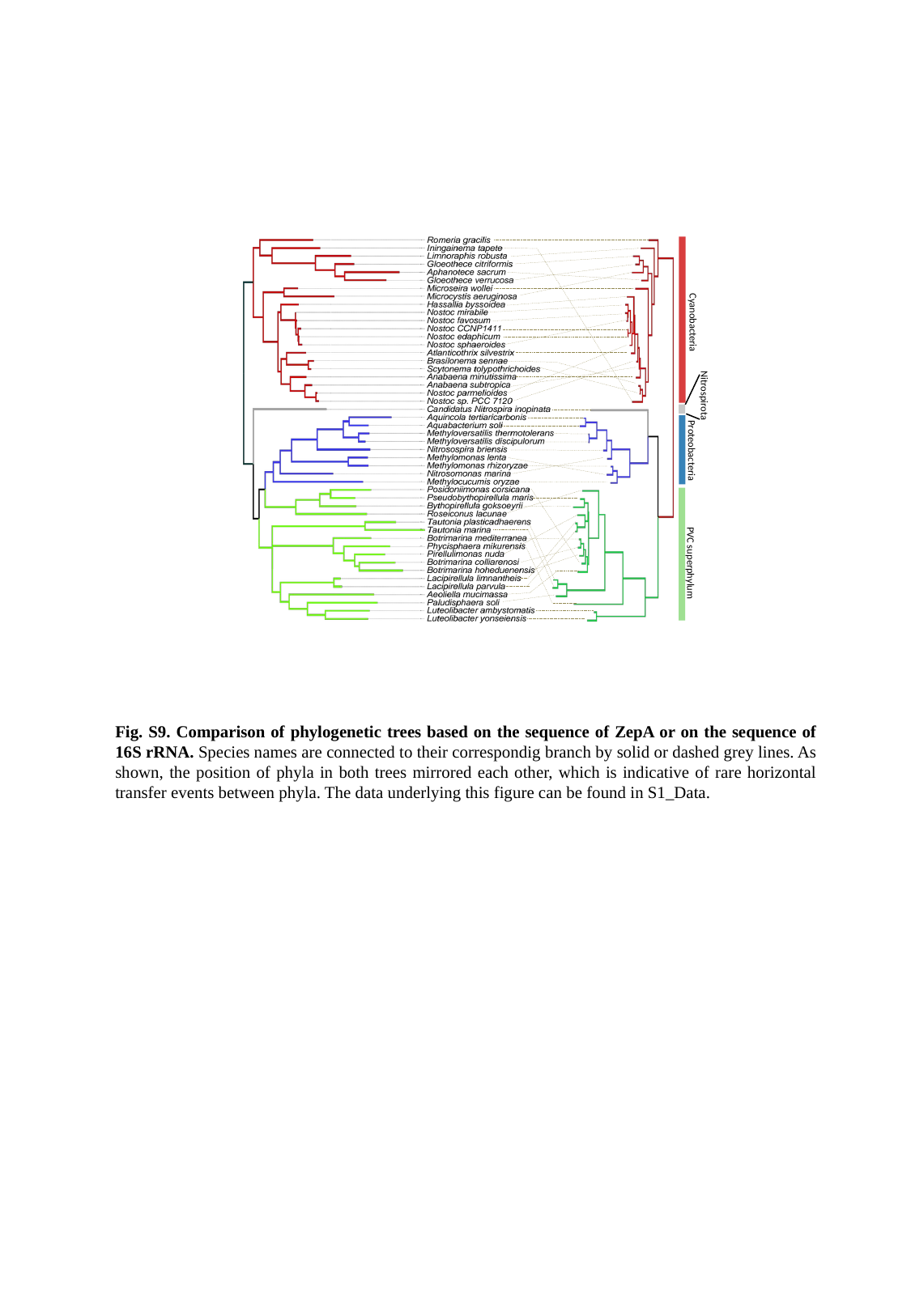

Cyanobacteria
Nitrospirota
Proteobacteria
PVC superphylum
Fig. S9. Comparison of phylogenetic trees based on the sequence of ZepA or on the sequence of 16S rRNA. Species names are connected to their correspondig branch by solid or dashed grey lines. As shown, the position of phyla in both trees mirrored each other, which is indicative of rare horizontal transfer events between phyla. The data underlying this figure can be found in S1_Data.
