# Supplementary material for: An ancient bacterial zinc acquisition system identified from a cyanobacterial exoproteome
Source: PLoS Biol. 2024 Mar 11;22(3):e3002546. doi: 10.1371/journal.pbio.3002546 (PMC10957091; doi:10.1371/journal.pbio.3002546)
Supplement: S10 Fig — Plates in Fig 3 and 2 biological replicates of this experiment were photographed and growth in each spot was quantitated using ImageJ software as indicated in Materials and methods. A Growth Index (GI) was defined as the sum of the density of all spots of one particular strain in a plate. This index is indicative of the capacity of each strain to grow in a particular medium. Colors in the heatmaps correspond to the average GI for each strain and condition according to the scale shown on the left. Darker color indicates a higher growth capacity. For statistical analysis, the average GI of a mutant strain in one condition was compared to the average GI of the wild type in the same condition using the Student’s t test. * Indicates p < 0.05, ** indicates p < 0.01, not significant differences are not indicated. The data underlying this figure can be found in S2 Data. (PPTX) [file pbio.3002546.s010.pptx]

## Slide 1
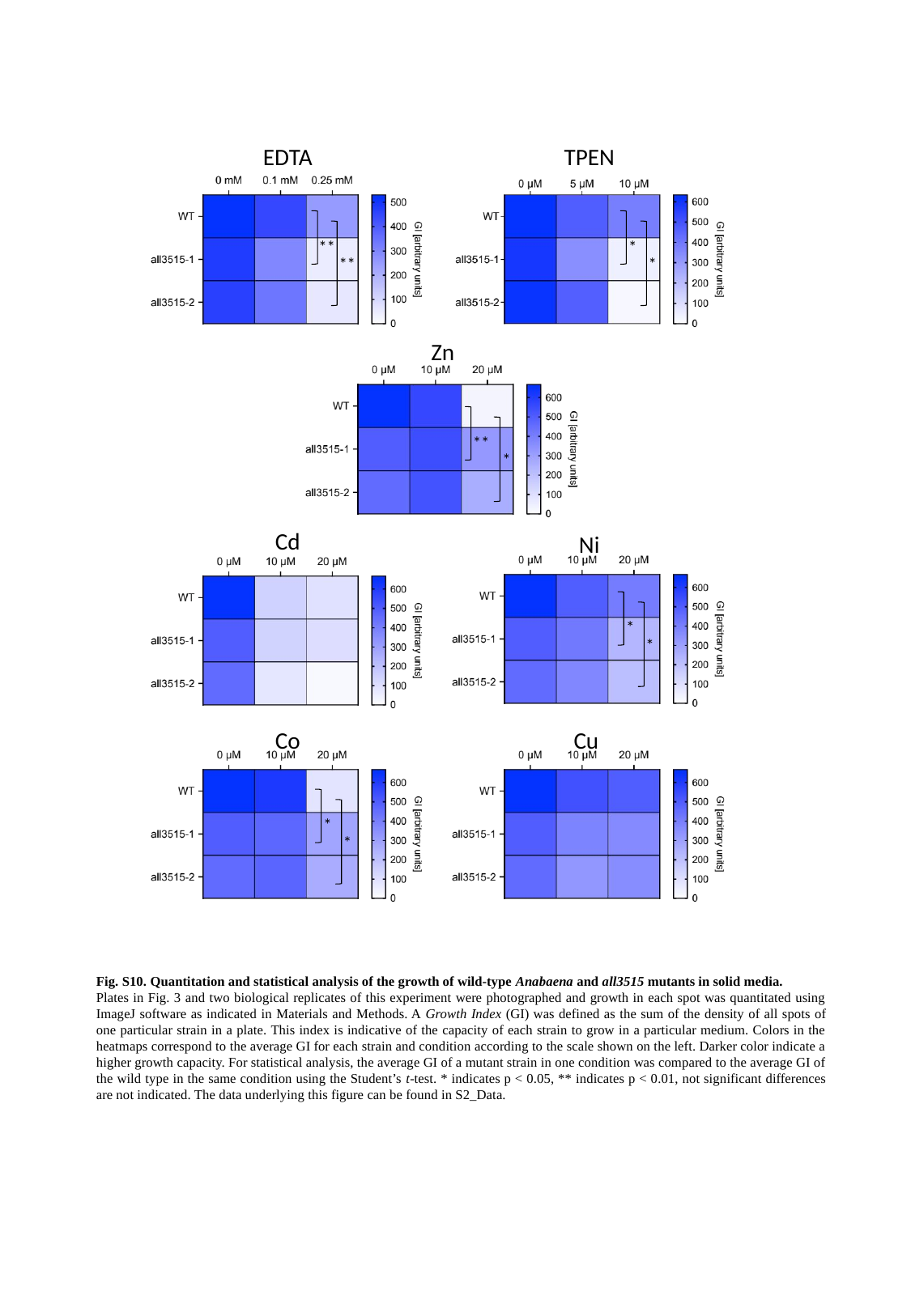

TPEN
EDTA
**
**
*
*
Zn
**
*
Cd
Ni
*
*
Co
Cu
*
*
Fig. S10. Quantitation and statistical analysis of the growth of wild-type Anabaena and all3515 mutants in solid media.
Plates in Fig. 3 and two biological replicates of this experiment were photographed and growth in each spot was quantitated using ImageJ software as indicated in Materials and Methods. A Growth Index (GI) was defined as the sum of the density of all spots of one particular strain in a plate. This index is indicative of the capacity of each strain to grow in a particular medium. Colors in the heatmaps correspond to the average GI for each strain and condition according to the scale shown on the left. Darker color indicate a higher growth capacity. For statistical analysis, the average GI of a mutant strain in one condition was compared to the average GI of the wild type in the same condition using the Student’s t-test. * indicates p < 0.05, ** indicates p < 0.01, not significant differences are not indicated. The data underlying this figure can be found in S2_Data.
