# Supplementary material for: An ancient bacterial zinc acquisition system identified from a cyanobacterial exoproteome
Source: PLoS Biol. 2024 Mar 11;22(3):e3002546. doi: 10.1371/journal.pbio.3002546 (PMC10957091; doi:10.1371/journal.pbio.3002546)
Supplement: S1 File — Palindromic sequences found enriched in the regions below by the MEME program (https://meme-suite.org/meme/tools/meme) are highlighted in yellow. The palindromic sequence in the upstream region of the zepA gene from Luteolibacter yonseiensis is highlighted in green. Promoter sequences (−35 and −10 boxes) predicted by BPROM (http://www.softberry.com/berry.phtml?topic=bprom&group=programs&subgroup=gfindb) are shown in red. The last nucleotide of each sequence corresponds to the −1 position. (DOCX) [file pbio.3002546.s011.docx]

**File S1. Palindromic sequences enriched in the upstream region of *zepA* genes.** Palindromic sequences found enriched in the regions below by the MEME program (https://meme-suite.org/meme/tools/meme), are highlighted in yellow. The palindromic sequence in the upstream region of the *zepA* gene from *Luteolibacter yonseiensis* is highlighted in green. Promoter sequences (-35 and -10 boxes) predicted by BPROM (http://www.softberry.com/berry.phtml?topic=bprom&group=programs&subgroup=gfindb) are shown in red. The last nucleotide of each sequence corresponds to the -1 position.

**CYANOBACTERIA**

>Anabaena_7120 637233905 hypothetical protein [Nostoc sp. PCC 7120: NC_003272] (-)strand

CGTGGTGGAAACTCTTAACAGAAGGGAAGATGAGCATCACAAGCAAGGTTTAAAGCCGAGTGGAATTGTAGGCAGCTTAGTTTCCCTGTTCCCTTTCATACTGGATGGATATTTGCTTCTTAATAAATTCTTCATTGACAGCCCCTTGCTGATTATGATAATCATTATCGGAAGTAAGTATACTGTCAGAAATAAGTGAT

>Leptolyngbya_BC1307 2883215987 hypothetical protein [Ga0442178_020 : Leptolyngbya sp. BC1307] (+)strand

GCGGCTGTAGTTTGAAGAAGCTGTGAAGATTGTCCTGTCTCCAGAACAGAACCTCAGAGGAAGTATTGAATTCATGATATTTTTGAGAATGATAATCATTATCGTATTATCGCAAAATCACTCAGGAATTCAATATGCTTTTCTTTAAGTCGCTGCTTGCCAGCGCTCTGTTTGCACTTCCTTTAAGTTTGACGGGGATT

>Brasilonema_UFV-OR1 2886766158 hypothetical protein [Ga0442302_287 : Brasilonema octagenarum UFV-OR1] (-)strand

TTTTAATGACGACTTGTTGCTGAATAAGATTCTGGAAAAATAACAAGAGAATGATGGTATTTTTTATACACTTAATTATTAGCACTCTAGCGCAAAAGAATAGCTTGAGCATTTCTTTATAAATTCTTAATGTTTGGAAAATCAAATATTATGATAATCATTATCATAAACTAGAAATTACATTCAAATGGAGAAGCGTG

>Microcystis_2520 2887290506 hypothetical protein [Ga0440941_040 : Microcystis aeruginosa NIES-2520] (-)strand

GGCCTCATCCCCAGAGAACCGGCTGCTGTTCTGGCGTTGCCTGTTCCTTGATCCACCTCGACCCCTTTAGATGCTCGGGCAACTTATTGGGACAGCAGTGTTCATGTTAACTTAAATTAACTTTGTTGGCAAAAGTGAATCGGAACAAGGGTTTTTCTCTAAAATGATAATGATTATCGTTCTAATTAAGGAGAGAAATC

>Anabaena_FACHB-250 2909468290 hypothetical protein [Ga0477596_098 : Anabaena minutissima FACHB-250] (-)strand

ATATTTTTGTGTGAATTTGCCATTAATGCAAGCTGCAATCTACACTATTTGCTGTTTTTGGCATCTGTACTTAGTCATAAATATTCACAATTCACCAGGATTGAGGACTTCTATTTTTAACAAATTCTTCATTGACAAACCCATAAAGAGCATGATAATCATTATCGTTGGTAAAAATTATACTTTCAGGGAGAACCCTA

>Nostoc_FACHB-3921 2913873837 hypothetical protein [Ga0477576_083 : Nostoc parmelioides FACHB-3921] (-)strand

CGTGGTGGAAACTCTTAACAGAAGGGAAGATGAGCATCACAAGCAAGGTTTAAAGCCGAGTGGAATTGTAGGCAGCTTAGTTTCCCTGTTCCCTTTCATACTGGATGGATATTTGCTTCTTAATAAATTCTTCATTGACAGCCCCTTGCTGATTATGATAATCATTATCGGAAGTAAGTATACTGTCAGAAATAAGTGAT

>Anabaena_FACHB-170 2914216792 hypothetical protein [Ga0477581_29 : Anabaena cylindrica FACHB-170] (+)strand

CGTGGTGGAAACTCTTAACAGAAGGGAAGATGAGCATCACAAGCAAGGTTTAAAGCCGAGTGGAATTGTAGGCAGCTTAGTTTCCCTGTTCCCTTTCATACTGGATGGATATTTGCTTCTTAATAAATTCTTCATTGACAGCCCCTTGCTGATTATGATAATCATTATCGGAAGTAAGTATACTGTCAGAAATAAGTGAT

>Microcystis_T1-4 2533923689 PEP-CTERM protein-sorting domain-containing protein [Microcystis sp. T1-4 : CAIP01000232] (-)strand

GGCCTCATCCCCAGAGAACCGGCTGCTGTTCCAGCGTTGCCTGTTCCTTGATCCACCTCGACCCCTTTAGATGCCCGGGCAACTTATTGGGACAGCATTGTTCACGTTAACTTAAATTAACTTTTTTGGCAAAAGTCAATCGCAACAAGGGTTTTTCGCTAAAGTGATAATGATTATCATTCCAATTAATGAGAGAAATC

>Microcystis9717 2535018890 PEP-CTERM protein-sorting domain-containing protein [Microcystis aeruginosa PCC 9717 : CAII01000439] (-)strand

GGCCTCATCCCCAGAGGACCCGCTGCTGTTCCAGCGTTGCCTGTTCCTTGATCCACCTCGACCCCTTTAGATGCTCGGGCAACTTATTGGGACAGCATTGTTCACGTTAACTTAAATTAACTTTGTTGGCAAAAGTGAATCGGAACAAGGGTTTTTCGCTAGAATGATAATGATTATCGTTCTAATTAAGGAGAGAAATC

>Microcystis9701 2535021094 PEP-CTERM protein-sorting domain-containing protein [Microcystis aeruginosa PCC 9701 : CAIQ01000009] (-)strand

GATCAAGATAAACTTTGACGGGATTTGGCCTCATCCCCAGATAACCTGCTGCTGTTCCGGCGTTGCCTGTTGGCCCGGGCAACTTATTCCGACAGCATGGTTCACGTTAACTTAAATTAACTTTGTTGGCAAAAGTGAATCAGAACAAGGGTTTTTCGTTAATGTGATAATGATTATCATTCTAATTAACGAGAGAAATC

>Microcystis2549 2645935751 PEP-CTERM protein-sorting domain-containing protein [Microcystis aeruginosa NIES-2549 : Ga0078274_11] (+)strand

GATCAAGATAAACTTTGACGGGATTTGGCCTCATCCCCAGATAACCTGCTGCTGTTCCGGCGTTGCCTGTTGGCCCGGGCAACTTATTCCGACAGCATGGTTCATGTTAACTTAAATTAACTTTGTTGGCAAAAGTGAATCAGAACAAGGGTTTTTCGTTAATGTGATAATGATTATCATTCTAATTAACGAGAGAAATC

>Microcystis2481 2722760219 PEP-CTERM protein-sorting domain-containing protein [Microcystis aeruginosa NIES-2481 : Ga0175438_11] (+)strand

GATCAAGATAAACTTTGACGGGATTTGGCCTCATCCCCAGATAACCTGCTGCTGTTCCGGCGTTGCCTGTTGGCCCGGGCAACTTATTCCGACAGCATGGTTCATGTTAACTTAAATTAACTTTGTTGGCAAAAGTGAATCAGAACAAGGGTTTTTCGTTAATGTGATAATGATTATCATTCTAATTAACGAGAGAAATC

>NostocKVJ20 2776639572 predicted secreted protein with PEP-CTERM sorting signal [Nostoc sp. KVJ20 : Ga0186886_1372] (+)strand

AGAGGAGCGTTGGGCAGTTATCCTCCTGCCTCCTGCCCTCTGCCTCCTGCCTTCCTCGATAAATATTCACAAGTTGGCGTAGCTGATAAGAATATTCCAACTCACCCTGCGGGAAGTCGCAGGTTAATACCTTGACAATGCCACAACAGGCATGATAATCATTATCACATAGAAAAATTATAGATTGATGGAGAAGCGTG

>Chlorogloea695 2789942002 predicted secreted protein with PEP-CTERM sorting signal [Chlorogloea sp. CCALA 695 : Ga0303536_1077] (-)strand

ATTAGCGAAACTAAGCTGTTTTGATTGGGGGCGATCGCATCTTCCGCCAACTTTTACTTGACATCTATGTTTGAGAATGATAATCATTATTTAAACGAGTAAGGATTTATCTGTGCCTGCACAAATTGTTGATTGGCATAGACCTTATAAATTTGCTGGTTTGAGTACCCATTAATTTGTTCAAACATGGAGACTAGATA

>NostocCENA543 2791300020 predicted secreted protein with PEP-CTERM sorting signal [Nostoc sp. CENA543 : Ga0272299_13] (+)strand

TAATACCAAATTGAGATAGGTAGTATTGTTTCGGAGTTTTGGAATAGCCTAAGAATCTAGGCTTTTAATCTAAAATCCAAAATCTAAAATCCAAAATTGGTACAGGCTTCTACTTTTTAACAAATTCTTCATTGACAAGCAAGTCGATAAAATGATAATCGTTATCACTAGCAAAAATTATACTTTAATGGAGAAACCCA

**PROTEOBACTERIA**

>Methylov_FAM1 2517051657 PEP-CTERM protein-sorting domain-containing protein [Methyloversatilis sp. FAM1 : MetFAM1DRAFT_chromosome1.1] (+)strand

CCCCGGAAAGAACAGCCGGCTACAGGCCGGCATGCCTGCCCGCCCTCCTCTTCTTTCCCCCAACGCTGCGTTCCGCCCCGCCTCGTCCGCCGCGTACCGCGGCCTATACGGCAGCACCCCGCATTTGATAATCCATTATCGTTTACGGGCACCGCAGGAGCGGCTACTATTGCGACTCGATTATCATCTGGAGAACTGAA

>Rhodocyclaceae bact RZ94 2517238006 PEP-CTERM protein-sorting domain-containing protein [Rhodocyclaceae bacterium RZ94 B594DRAFT_scaffold1.1] (+)strand

CCCCGGAAAGAACAGCCGGCTACAGGCCGGCATGCCTGCCCGCCCTCCTCTTCTTTCCCCCAACGCTGCGTTCCGCCCCGCCTCGTCCGCCGCGTACCGCGGCCTATACGGCAGCACCCCGCATTTGATAATCCATTATCGTTTACGGGCACCGCAGGAGCGGCTACTATTGCGACTCGATTATCATCTGGAGAACTGAA

>Methylov_RZ18-153 2517353217 PEP-CTERM protein-sorting domain-containing protein [Methyloversatilis sp. RZ18-153: MetRZ18153DRAFT_Scaffold1.1] (-)strand

CCGATGGTGACGTCGAAGTGGATGGCATGTGCCGCGGCAACGTGGAAGGACAGCAGTGCTGCAGCAAGGTGCATGAAGCGGATGGGCACGCGCATGAGAGGTCTCCGGATGATCAGCCTCTAATGATAATGCAATCTCCTTTACAAAAAAGACTACGCCCCGTATCTTTTGATATTCGATTATCAACAAAGGAGAGCATC

>Methylov_NVD 2517417617 PEP-CTERM protein-sorting domain-containing protein [Methyloversatilis sp. NVD : A3Q7DRAFT_scaffold1.1] (+)strand

AGCAAGGACATGGAAATCTCCGTGGCTTGATTTTTGATAACGCACTACCATTTGCAAAAATAACAGCATTGGGACAGGACGTACAGCGTCGGCGGACCTTGCCGGCCTTGATCGAAAGAGTTTGTGCGCTCTCTATTGATAATTTATTATCGTTAGTGGCTGGCCAGCCATCTTTCCAATCCCTCATCCAGGAGACACAG

>Nitrosospira_C-128 2588204682 PEP-CTERM protein-sorting domain-containing protein [Nitrosospira briensis C-128 : F822DRAFT_unitig_0_quiver_dupTrim_8202.1] (-)stran

AAAGAAATATGAGTGGCGGTAGAGGTTGAATGCCTGAGCTTAGCGCCACCGAAAAATCCCTTGAGCAATAATCGTTCTTGACCATACGCTATTGATAAACTATTATCAATAGCGTCACTCCCGTATAAGCTCGGCTTATGCATCAATGGCTACGATTTCATCTCACGACTTTATCTTACAACTGCACACGGAGAAAGTAT

>Nitrosospira_NpAV 2630434638 PEP-CTERM protein-sorting domain-containing protein [Nitrosospira sp. NpAV : Ga0077272_1021] (+)strand

TCGATTTCGGGATGGGTTGCTGGCGATATCAGCACAAGCAGCCCAATCTTTGGTTCATGGCATTAACAAGCCTTGACTATATGCTATTGATAACTTATTATCAATTAATCCATGTCGATCATGATATAGAAAAATTGGATCCTAATAGAGATGAAAGTAGTAGCACATGTTCATTTTATAACTGTAAAAGGAGAAAGTAT

>Nitrosovibrio_Nv17 2671308924 PEP-CTERM protein-sorting domain-containing protein [Nitrosovibrio sp. Nv17 : Ga0111729_117] (+)strand

CGCCGCATGCAATAAAAAAGGGCCAGCCTGCACAAAATGATCCCGCATCGCCTTGACTATATGCTATTGATAACTTATTATCAATAGCACCATGCATGCCGCTGGCGGCGGCCCGCCGATGACGGCAGGCGGCCACCACGCGCATTCGTGGCAAGAAACGTGGGCAGGGCCCATTTTATAACCGCAGAAGGAGAATGTAT

>Nitrosolobus_Nl7 2671429911 PEP-CTERM protein-sorting domain-containing protein [Nitrosolobus multiformis Nl7 :Ga0111719_103] (-)strand

GAAATAACGTCAAGAATTGCATCCCGCTGCCGAGTATTACGTTCCATGGATTTGCCTCAACAATATCGTCCCATATCTATATAGATATTATCCGATTCGCCAGCCTCTTAACTCCTATTATGTCATCAGCAGCCTTGACCGGATACTATTGCTATTGATATATTGTTATCATTAACAATAACTGAAGAAAGAGGAGATAG

>Nitrosospira_Nsp11 2671441400 PEP-CTERM protein-sorting domain-containing protein [Nitrosospira sp. 1 Nsp11 : Ga0111721_105] (+)strand

GATAAAAAGAAATATAAGTGGCGGTAGAGGTTGAATGCCTGAGTTTAACGCCGCCGAAAAATCCCTTGAGCAATAATCAGCCTTGACCATATGCTATTGATAAACTATTATCAATAGCGCGATTCCCCGGTATAAGCTCGGCTTATGCATTGGCAATGACACGACTACAATTTTATCTTACACCTACACATGGAGAAAGT

>Nitrosospira_Nsp13 2671446205 PEP-CTERM protein-sorting domain-containing protein [Nitrosospira sp. 5 Nsp13 : Ga0111725_101] (+)strand

ATCGATTTCGGGATTGGTTGCTCGCGATATCGGCACGGGCAACCCAATCTCCACTCATGGCATTAACAAGCCTTGACTATATGCTATTGATAACTTATTATCAATTATTCCATGCTGATCGTAGTATGGAAAAAATCGGATCCAATGGAGATGAAAGTTGTGTCATGCATTCATTTTATAACTGTAAAAGGAGAAAGTAT

>Nitrosolobus_Nl18 2671452574 PEP-CTERM protein-sorting domain-containing protein [Nitrosolobus multiformis Nl18 Ga0111717_121] (-)strand

GAAATGACATCAAGAATTGCATCCCGCTGCCGAGTATTACGTTCCATGGATTTGCCTCAACAATATCATCCCATATCTATATAGATATTATCCGATTCGCAAGCCTCTTAACTCCTATTATGTCACTAACAGCCTTGACCGAATACTATTGCTATTGATATATTGTTATCACTAACAATAACTGAAGAAAGAGGAGATAG

>Nitrosospira_Nsp1 2671455122 PEP-CTERM protein-sorting domain-containing protein [Nitrosospira sp. 1 Nsp1 : Ga0111720_117] (+)strand

CACACGCTACTGATAAACTATTATCAATAGCGTTGCTCCCATATAAGCTCGGCTTATGCATCGATAGCCACGATTTTATCTCACAGCTTTATCTTACAACTGCACACGGAGAAAGTATATGCGTTCCAACGCGTTATATGCCGGCTTGGCTGGCTGGGCTATTATGATGGCTTCCTTCAATATCTCGGCATCATCACGGC

>Nitrosolobus_Nl14 2671462041 PEP-CTERM protein-sorting domain-containing protein [Nitrosolobus sp. Nl14 : Ga0111716_121] (+)strand

TGAAATGACGTCAAGAATTGCATCCCGCTGCCGAGTATTACGTTCCATGAATTTGCCTCAACAATATCATCCCATATCTATATAGATATTATCCGATTCACGAGCCTCTTAACTCTCATTATGTCATCGTAGCCTTGACCGAATACTGTTGCTATTGATATATTATTATCACCAAAAATAACTGAAAAAAGAGGAGATAG

>Nitrosospira_Nsp18 2671463504 PEP-CTERM protein-sorting domain-containing protein [Nitrosospira sp. 1 Nsp18 : Ga0111722_107] (-)strand

GATAAAAAGAAATATAAGTGGCGGTAGAGGTTGAATGCCTGAGTTTAACGCCGCCGAAAAATCCCTTGAGCAATAATCAGCCTTGACCATATGCTATTGATAAACTATTATCAATAGCGCGATTCCCCGGTATAAGCTCGGCTTATGCATTGGCAACGACACGACTACAATTTTATCTTACACCTACACATGGAGAAAGT

>Methyloversatilis_RAC08 2721493003 PEP-CTERM protein-sorting domain-containing protein [Methyloversatilis sp. RAC08 : Ga0175218_11] (-)strand

GGATGGCGTGGGCCGACACGGCATGCAGCATCAGCAGGCCGAAGGCAAGTCGGGGCAGGACGGACATGGCGGGAACTCCGAGCGAGACGATAGCTGATGATAACGCATTGCTATTTACAACAGTGCGCAAGCGGCTTATCTTGATAATACGTTATCGTTAGTGAATGCGCATCCCACCCCACCCCTGAAGGAGCTGTTTC

>Nitrosospira_Nl1 2737462508 predicted secreted protein with PEP-CTERM sorting signal [Nitrosospira multiformis Nl1 : Ga0181046_103] (+)strand

TGAAATGACGTCAAGAATTGCATCCCGCTGCCGAGTATTACGTTCCATGAATTTGCCTCAACAATATCATTCCATATCTATATAGATATTATCCGATTCACGAGCCTCTTAACTCTCATTATGTCATCGTAGCCTTGACCGAATACTGTTGCTATTGATATATTATTATCATTCAAAATAACTGAAAAAAGAGGAGATAG

>Nitrosospira_Nl2 2737466245 predicted secreted protein with PEP-CTERM sorting signal [Nitrosospira multiformis Nl2: Ga0181048_105] (-)strand

TGAAATGACGTCAAGAATTGCATCCCGCTGTCGAGTATTACGTTCCATGAATTTGCCTCAACAATATCATCCCATATCTATATAGATATTATCCGATTCACGAGCCTCTTAACTCTCATTATGTCATCGTAGCCTTGACCGAATGCTATTGCTATTGATATATTATTATCATCAACAATAACTGAAGAAAGAGGAGATAG

>Nitrosospira_Nsp22 2737532361 predicted secreted protein with PEP-CTERM sorting signal [Nitrosospira sp. Nsp22 : Ga0181056_102] (+)strand

AAAGAAATATGAGTGGCGGTAGAGGTTGAATGCCTGAGCCTAGCGCCGCTGAAAAATCCCTTGAGCAATAATCGTTCTTGACCATGCGCTACTGATAAATTATTATCAATAGCGTCACTCCCGTATAAGCTCGGCTTATGCATCGATGGCTACGATTTTATCTCACAACTTTATCTTACAACTGCACACGGAGAAAGTAT

>Nitrosospira_Nl3 2737557987 predicted secreted protein with PEP-CTERM sorting signal [Nitrosospira multiformis Nl3: Ga0181069_106] (-)strand

TGAAATGACGTCAAGAATTGCATCCCGCTGTCGAGTATTACGTTCCATGAATTTGCCTCAACAATATCATCCCATATCTATATAGATATTATCCGATTCACGAGCCTCTTAACTCTCATTATGTCATCGTAGCCTTGACCGAATGCTATTGCTATTGATATATTATTATCATCAACAATAACTGAAGAAAGAGGAGATAG

>Nitrosomonas_HPC101 2898508631 hypothetical protein [Ga0443776_08 : Nitrosomonas sp. HPC101] (+)strand

TCTGGCAAGGATCTATTCAGTTACAGTCCGTTCTTCCAATTTACATAGCTTGTCTTGTATTCGAATCTTGACTGCAATGCTATTGATATCTTATTATCAATAGCCGTTTGTTCTGCTTCACGGCTATGGCGTCTGCTCATTCGAGCAAGGGCAGAGGTCTCAGCGAAAGCCTATTTTATAATTGCAGAAGGGAAATGTAT

**PVC SUPERPHYLUM**

>Luteolibacter_JCM 18052 2994579025 hypothetical protein [Ga0487610_10] (-)strand Luteolibacter yonseiensis JCM 18052

CAGCAACTCCAACAGGGTGAAACCGCGACGCAGGCGTCGCGGAACGATTCGGGGACATTTCAACGGGCTCACGCCGAAAAATTAATACACAACATTTCCAAATGCAATATTATTGCATTTGGAAATACATGGCATTAGTGCTCTTGCATCTCGCGGAGCCCCACGAGGCCGAGCGGGAGCCCTGAAATCAATCCACACCC
