# Supplementary material for: An ancient bacterial zinc acquisition system identified from a cyanobacterial exoproteome
Source: PLoS Biol. 2024 Mar 11;22(3):e3002546. doi: 10.1371/journal.pbio.3002546 (PMC10957091; doi:10.1371/journal.pbio.3002546)
Supplement: S2 File — (DOCX) [file pbio.3002546.s012.docx]

**File S2. Extended Materials and Methods**

**Generation of specific antibodies against ZepA**

For the generation of antibodies against ZepA, an internal fragment of this protein was overexpressed in *E. coli* and purified. In order to this, plasmid pCi21 was constructed by PCR amplification of *Anabaena* genomic DNA with primers ALL3515-7F and ALL3515-6R and the resulting fragment was cloned in the pET28b vector (EMD Biosciences) after digestion with NdeI and XhoI. pCi21 was introduced in BL21(DE3) and overexpression of the protein was induced by culture for 3 hours in liquid LB medium in agitated flasks at 37ºC by addition of 1 mM IPTG. After disruption of cells by three passages through a French press, inclusion bodies were isolated by centrifugation for 20 min at 15000 x *g* followed by two washes in a buffer containing 100 mM Tris pH 8, 150 mM NaCl, 1 mM EDTA and 1% (v/v) Triton X-100. The pellet was resuspended in a buffer containing 100 mM Tris pH 8, 150 mM NaCl, 1 mM EDTA, 2% (w/v) N-lauryl sarcosine, 2% (v/v) Triton X-100 and 0.1% (w/v) CHAPS (3-((3-cholamidopropyl) dimethylammonio)-1-propanesulfonate). The resulting suspension was centrifuged for 20 min at 15000 x *g* and the supernatant was loaded in preparative SDS-PAGE gels that were stained by immersion in 0.3 M CuCl_2_. A major band of ca. 30 KDa was excised and sent to Covalab (<https://www.covalab.com/eu/>) for generation of antibodies by rabbit injection.

**Western blot**

Western blots were performed by transfer of proteins resolved by SDS-PAGE to Hybond-P PVDF membranes (Amersham) and incubation in the presence of anti-ZepA antisera diluted 1:1000 in TBS buffer (100 mM Tris pH 7.5, 500 mM NaCl) supplemented with 0.1% (v/v) Tween-20 and 5% non-fat dried milk. Membranes were washed with TBS buffer supplemented with 0.1% (v/v) Tween-20 and incubated with an anti-rabbit secondary antibody coupled to horseradish peroxidase (Bio-Rad). After washing with TBS buffer supplemented with 0.1% (v/v) Tween-20 and a final wash with TBS buffer, westerns were revealed by incubation with the WesternBright ECL reagent (Advansta) and were visualized in an Amersham ImageQuant 800 equipment.

**Functional test of *Anabaena* ZepA and ZepA homologs**

Plasmids for ectopic expression of ZepA protein from distinct species were constructed as follows. A plasmid for expression of *Ananabaena* ZepA (pET28b:ZepA_Ana) was constructed by PCR amplification of the *zepA* gene from *Anabaena* with primers ALL3515-3Fb and ALL3515-3R. The resulting fragment was digested with NcoI and XhoI and cloned in the pET28b vector (EMD Biosciences) digested with the same enzymes. Plasmid pET28b:ZepA_Ana was used as a template for PCR with divergent primers 38DeltaStrep-F and 38DeltaStrep-R and used for the cloning by Gibson assembly of synthetic DNA fragments containing the coding sequence for the predicted mature ZepA protein from *Candidatus Nitrospira inopinata, Aquincola tertiaricarbonis* or *Lacipirellula limnantheis*. Synthetic DNA fragments containing the predicted mature sequence of these proteins optimized for the codon usage of *E. coli* were supplied by IDT (<https://eu.idtdna.com>) (Table S3). In the resulting plasmids, named pET28b:ZepA_Nitrosp, pET28b:ZepA_Aquinc and pET28b:ZepA_Lacip, respectively, the predicted sequence of the mature ZepA protein from the corresponding species was in frame with the N-terminal signal peptide and the PEP-CTERM domain of *Anabaena*. These plasmids were introduced in the *E. coli* strain Lemo21(DE3) (WAGNER, 2008 PNAS). Control strains contained the empty pET28b vector or the pET28b expressing GFP (pCi14 plasmid). For the construction of this plasmid, the coding sequence of the GFP-mut2 protein was amplified by PCR with primers GFPmut2-2F and GFPmut2-4R and cloned between the NcoI and BamHI sites of pET28b. Growth tests were performed in plates with M9 minimal medium with supplements (see Fig. 8) that were incubated at 37ºC.

**Fractionation of extracts of *E. coli* Lemo21(DE3) expressing *Anabaena* ZepA**

Protein overexpression and extract fractionation was performed following the protocol in the IBA-Lifesciences webpage (<https://www.iba-lifesciences.com/media/ae/aa/89/1689775604/Protocol%20Expression%20of%20recombinant%20proteins%20in%20E.%20coli.pdf>) with modification as follows. Flasks containing 100 ml of LB medium were inoculated with 5 ml of an overnight culture of E. coli Lemo21(DE3) containing plasmid pET28b:ZepA_Ana and incubated 1.5 hours at 37ºC in agitation (200 r.p.m.). IPTG was then added at a final concentration of 1 mM and the culture was further incubated for 3h at 37ºC in agitation. Cells were harvested by centrifugation at 8000 x *g* for 5 min at 4ºC. The supernatant containing the extracellular medium was lyophilized, resuspended in 5 ml of 100 mM pH 8, 150 mM NaCl, subjected to gel filtration through a preparative PD-10 column (GE Healthcare) for salt removal and concentrated 10-fold through a Vivaspin-500 device. The pellet was resuspended in 1 ml of buffer containing 100 mM Tris pH 8, 500 mM sucrose and 2 mg/ml polymyxin B sulfate (Merck) and incubated on ice for 30 min. The suspension was centrifuged for 5 minutes at 13000 x *g* at 4ºC and the supernantant containing the periplasmic material was saved. The pellet containing the spheroplasts was resuspended in 100 mM Tris pH 8, 500 mM sucrose.

**Overexpression and purification ZepA-StrepTag-II**

Plasmid pCV2 was constructed by Gibson assembly of pET28b digested with NcoI and HindIII with a synthetic DNA fragment supplied by Thermo Scientific containing the coding sequence of the signal peptide of the OmpA protein from *E. coli* fused in frame to the coding sequence of the predicted mature ZepA from *Anabaena* modified for optimization of the codon usage of *E. coli*, and fused in frame with the coding sequence of a HRV3C protease site and a StrepTag-II (Table S3). Plasmid pCV2 was introduced in *E. coli* Lemo21(DE3). Overexpression assays in the resulting strain demonstrated that the OmpA signal peptide drove the exportation to the periplasm of the ZepA-StrepTag-II protein, which accumulated in this compartment to a high level. A 3 liter culture of the pCV2-containing strain was induced with 1 mM IPTG, culturing under agitation (200 r.p.m.) at 37ºC and harvested by centrifugation at 5000 x *g* for 10 min at 4ºC. The pellet was resuspended in 30 ml of 100 mM Tris pH 8, 500 mM sucrose, 2 mg/ml polymixin B sulfate (Merk) and 1 mM PMSF (phenylmethylsulfonyl fluoride) and incubated on ice for 30 minutes. The suspension was subsequently centrifuged at 13000 x *g* for 5 minutes at 4ºC and the supernatant was supplemented with NaCl to a final concentration of 150 mM and loaded on a StrepTrap XT column (Cytiva) for ZepA-StrepTag-II purification following the instructions of the manufacturer.

**Analytical gel filtration chromatography**

Analytical gel filtration chromatography was performed using a Superdex 200 Increase 10/300 GL column (GE Healthcare) and 100 mM phosphate buffer pH 7.4, 140 mM NaCl as chromatography buffer in a NGC Chromatography System (Bio-Rad) coupled to a NGC fraction collector (Bio-Rad). The column was calibrated with the following MW standards vitamin B12 (1355 Da), ribonuclease A (13700 Da), chymotrypsinogen A (25000 Da), ovoalbumin (43000 Da), albumin (67000 Da), ferritine (440000 Da) and tyroglobulin (669000 Da). The void volume of the column was determined by passage of Blue Dextran 2000. The Kav of each protein was calculated according to the following equation Kav=(Ve-Vo)/(Vt-Vo), whereby Ve is the elution volume for each protein, Vo the void volume of the column and Vt the total volume of the column. The linear function defined by plotting the Kav against the logarithm of the molecular weight of each protein was used for the determination of the molecular weight of ZepA-StrepTag-II. 0.033 mg of pure ZepA-StrepTag-II protein was loaded in the column for determination of its molecular weight.

**ICP-OES**

1.5-1.8 mg of pure ZepA-StrepTag-II protein was subjected to preparative gel filtration chromatography through a PD-10 column (GE Healthcare) using 100 mM Tris pH 8, 150 mM NaCl buffer previously demetallated by mixing with 20 g/l BT Chelex 100 resin under stirring. Fractions of 1 ml were colected, frozen and sent for metal determination by ICP-OES to the Servicio de Ionómica (CEBAS-CSIC, Murcia, Spain). The concentration of protein in each fraction was determined using the Bradford reagent from Bio-Rad.
