# Supplementary material for: An ancient bacterial zinc acquisition system identified from a cyanobacterial exoproteome
Source: PLoS Biol. 2024 Mar 11;22(3):e3002546. doi: 10.1371/journal.pbio.3002546 (PMC10957091; doi:10.1371/journal.pbio.3002546)
Supplement: S3 Table — The position of putative Zur-binding sites respect to transcription start sites (TSSs) is depicted at the right side. “TSS not mapped” indicates that no TSS has been reported at a distance less than 500 bp from the Zur-binding site. (DOCX) [file pbio.3002546.s015.docx]

| **Gene** | **Zur-binding site** | **FIMO**  **p-value** | **FIMO**  **q-value** | **Matched Sequence** | **Localization** |
| --- | --- | --- | --- | --- | --- |
| *alr3242* |  | 4.2e-09 | 3.29e-05 | TGATAATCATTATCA |  |
| *all3515* |  | 2.31e-06 | 0.00452 | TGATTATGATAATCA |  |
|  |  | 1.43e-07 | 0.000559 | TGATAATCATTATCG |  |
| *alr4028*  *-4029* |  | 3.05e-07 | 0.000797 | TGATAATAATAATCA |  |
|  |  | 7.77e-05 | 0.0761 | TAATAATAATCATTA |  |
|  |  | 5.58e-06 | 0.00875 | TAATAATCATTATCT |  |
| *all0833* |  | 6.7e-06 | 0.00875 | TGAGAATTATTATAA |  |
|  |  |  |  |  |  |
| *all1691* |  | 4.46e-05 | 0.0236 | TGTTAATAATAAGCA |  |
|  |  | 7.42e-05 | 0.032 | TAATAAATATTCTCA |  |

**Table S3. Zur binding sites in the 5' upstream region of genes encoding O- and U-proteins.** The position of putative Zur-binding sites respect to transcription start sites (TSS) is depicted at the right side. "TSS not mapped" indicates that no TSS has been reported at a distance less than 500 bp from the Zur-binding site.
