# Supplementary material for: An ancient bacterial zinc acquisition system identified from a cyanobacterial exoproteome
Source: PLoS Biol. 2024 Mar 11;22(3):e3002546. doi: 10.1371/journal.pbio.3002546 (PMC10957091; doi:10.1371/journal.pbio.3002546)
Supplement: S4 Table — (DOCX) [file pbio.3002546.s016.docx]

**Table S4. Synthetic DNA used in this work**.

| ALL3515-2F | CAATATTGCTCGAGCGTAAGC |
| --- | --- |
| ALL3515-2R | GTACCAGGATCCAGCTAGGG |
| ALL3515-3Fb | TATACCATGGCTTCATACAGCATTCAACTG |
| ALL3515-3R | GATACAACCTCGAGACAAAAAG |
| ALL3515 R | TAAGACTTCACACTTCCGATAATGATTATCATAATCGGAAGCCGTTCATT |
| ALL3515-7F | CTACATATGAATATTGCACCAGCGC |
| ALL3515-6R | GAACTCGAGTCAAAATGTACCAGACTC |
| ALL4725-GS-1F | CTACAACACAAAATGAACGGCTTCC |
| ALL4725-GS-1R | TAAGACTTCACAAGATTGATAACCATTATCATAATGGAAGCCGTTCATTT |
| ALL4725-GS-2R | TAAGACTTCACAAGATTGATAACCATTGGGGTAATGGAAGCCGTTCATTT |
| A7120-RNPB-1F | TTGGTAACAAACGTCCCAGA |
| A7120-RNPB-1R | TAAGCCGGGTTCTGTTCTCT |
| PRL287-1F | CGCCTTCTATGAAAGGTTGG |
| 38DeltaStrep-F | GTGCCTGAACCTTCAACAACTATTAGC |
| 38DeltaStrep-R | AGCTTGCGCTGGTGCAATATTGC |
| GFPmut2-2F | ATACCCATGGGTAAAGGAGAAG |
| GFPmut2-4R | GTTTCATGTGATCTGGGTATC |
| Gblock-*Candidatus Nitrospira inopinata* | ATATTGCACCAGCGCAAGCTCAGGCAGTTGAATATTATGTTGGAATTGATGGGCGGGCGGTTATTCCAACGGGTACTTACGCAGGACTGGACAATCCGAATTACGGCCGGTTGACATTCCTGTATGCACACCTAAATGAGACAGATCCCACGCGGAATCATTACCATGGGATTGGAGCATACACCTACAGTGGGCCTGCTGACGCGCCAGTGATTAACCCCACCAATGCGAACAACCGAATCCCCGAGACATACACTGCGCAGCCGCCTCTCCCGTTATTCCCCGGTGAGGGTGTACACGCCGGATTGTGGGTGAGTCGCCCCGTTCCTGGGCTCGAATACAGTCTCCTGGAGTGGGCTCCAACAGATCGGATCCGGGACGCAGAACTCGGTTCCCCGGAACACTACCTATTTCACAGTTCTGGGGGCCGCTGGACACAGAGTCTCGAAGGAACCCGACTCGCATTACAGACAGTCGGACTGACGCCGGGGCTACGTGTAATGGATCAGACAGGTACAACCATTTTTACGGGGCCAGGCCAAACTTATGTCTTGGGTGAAGGCGATCGTTTCTCTTTTTTCCCGACTTTCTACACTGTGCTAGCGGATGAGAGCCGCTATACAGCCTCTTTCCGGCTGATCGACTTGAACGGTGGTGCTGGTCACAGCGGGATTTTCCATTTTGATTTTCGCCCTGTGCCTGAACCTTCAACAAC |
| Gblock-*Aquincola tertiaricarbonis* | ATATTGCACCAGCGCAAGCTAGCGGAATTCGAGCCGCAGAAGGAATTGGCTACTATATCGGGGTCGACTCTCTGCAAACGGTAGCATCCGGCACCTTCGCTGGTCTAGCGAACCCAAACGCTGGTCGGTTGACTTTACTCTTGGATCACGGCAACCATTTCCACGGAATCGGCGCCTACAGCTATACAGGAACTGCGGCGGCTCCAGTGGTCCAACCCACATCCGCTAACAATCGTTTACCTGAGCCGTCCTCCCGGGTCGATGAGGCGACTAGCAGTATCGCCCTACAAGCTGGGAGCGGAGCCTATGCAGGTCGTTGGGTCAGTCAACCTTTGGCTGCAGGTGCCCCAGCAGCGGATTATAGCTACTTGGGGGGAGCAAGTATCCAATCCTTATCTGGACTGGGCACCGCAGCAGGTGTTCTATACAATTCCTCCGCCGGGCGCTGGAATGCTGCGTTTAGCGATGTTGTTGTTGGTCTCAAGTTGGAAAGTATTAGTAGCGGTTTAAAAGTCGCCATTGGAAATGAGATGGACGCTTTCGCTGCAGGGGTAGGGAGCGTTTTTACTCTCGGGGACAGTGGATCTCTAAACTTCTTGCCGACGTTTCATGCCGACGCTAACGTTGCTCCTGGAGTCTACAGCGCCCAGTTTTCCCTCGTCAACTTAGGCGGTAATTCTGCGGTTCAATCCGGCGGGTCTTTCGCTTATGACTTCTCCGTTGCAGCACCTGTGCCTGAACCTTCAAC |
| Gblock-*Lacipirellula limnantheis* | ATATTGCACCAGCGCAAGCTGCCGAGCACAATTATTATGTTACAGTAGACGGGCGCCCTACCTTAACTAGCGGAACTTATGCTGGTCTAGGCAATCCTAATAGCGGACGCCTCACACTCCTATATGCGCATTGGAATGACGATACGCCCTCTAGCAACCACTTTCATGGTATTGGAGTGTATAGCCTGACAGGGCCCGTCGATGCACCGATTGTAGCAGACACTAACGGGAATAACCGTCTCCCAGAGACCTATACTGGCCAGGCACCCCTCACTCTAAAGCCTGGCAGCGGAGCTTTCGCGGGTAAGCTCGTATCTGGGGAAAACGGGGAGCACTATTCCGATCTCTCCTTATACAGTATCCATGATCTCGCTGCCGCCGCCACTTTAAATCCGACCAGTCCGCAAGGATACATGTATAACTCCAATGCTGGTTACAAAAACACCCCAATGACTGGCTTGAACCTGGCCTTGGAAATCGTCAGCATCAGTCCTGGGCTCAACTTAGGTCAGGCCGGATTAAACCAGTCCGGAGATCGCCTAGCTATCGGAGGCGAGGCATCCTGGCCTTTCGAGCCAGTCTTCTGGACGGCAGAGAACGCCGCTCCTGGAAACTATAGCGCAAGTCTCCGCTTAGTAGACCAGGCTAACGTATTTGGTTCTAGTGGCACATTTCACGTTGATTTCGCTGTGGTGCCTGAACCTTCAACAAC |
| OmpA-ZepA-Strep | ACTTTAAGAAGGAGATATACATGAAAAAGACAGCTATCGCGATTGCAGTGGCACTGGCTGGTTTCGCTACCGTAGCGCAGGCCCACGGAAATCATACTCACGCTGATGAAACAGAGTTCTATATTGGTCTCGATGGCCTGCGTGTGCTGGCTAGCGGTACTTACGCTGGCTTGGACAACCCGAACTACAATAGATTGACCTTCCTGTATGCGCATCGTGAAGAAGATTTTACGACCAACCACTTTCACGGCATCGGAGCCTATAGCTACTCTGGTCCGCTGGGTTCTCCGTCCATCAACCCGACAAATATTAACAACCGCATCCCAGAAACGTACACCGGTCAGATTCCGCTGCAACTTCTGCCGGGTAAAGGCGCCTTTGCGGGTCGCCTCGTGTCTACCGCAACCGGTGCGGAGTACAGCAACATCAAAATCGAAACCGTTGAGACCCTGGCGAGCGCGACCGACGCTGATGATCAATATCTGTTCAACTCCTCCGGTGGTCGTTGGCAGAGCAGCCTGGGCGGTGCGACGATCGGCTTACAACTGATTTCGATTTCTAGCGGGTTGAATGTTGCAGACGAAGCAGGCGTTAATCTGTTTAACAGCGTGGGTGACATCTATACCATTGGCACCGGCGACAATTTCACCTTCCGTCCGAAATTCTGGACCGATGCGGCCGCGGCACTGGGCAAGTACTCAGCGACGTTTAAGCTGGTGGATGTTTCCACCGACAGCTCGACTCCGCTGTTGGAGAGCGGTACCTTCTCCTTTGACTTCAGCGTCGAGCAGGGTGCACTTGAAGTCCTCTTTCAGGGACCCGGTTGGAGCCACCCGCAGTTCGAAAAGTAAAGCTTGCGGCCGCACTCGAG |
