# Supplementary material for: An ancient bacterial zinc acquisition system identified from a cyanobacterial exoproteome
Source: PLoS Biol. 2024 Mar 11;22(3):e3002546. doi: 10.1371/journal.pbio.3002546 (PMC10957091; doi:10.1371/journal.pbio.3002546)
Supplement: S1 Raw Images — (PDF) [file pbio.3002546.s019.pdf]

Fig 1C Left panel

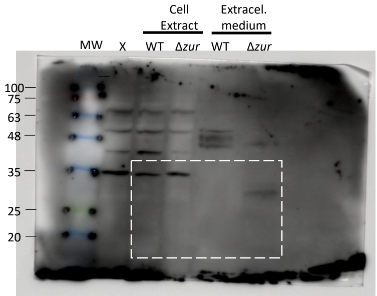

Image captured with an Amersham ImageQuant 800 equipment

Fig 1C Right panel

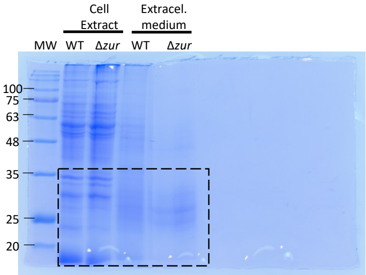

Image captured with a Canon EOS100D camera

Fig 2A Left panel

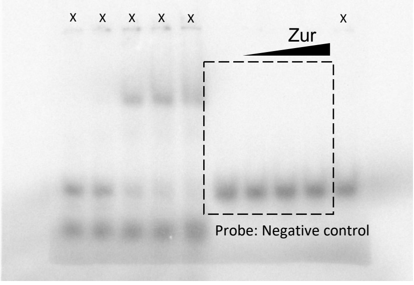

Image captured with a Cyclone Plus Phosphor System (Perkin Elmer)

Fig 2A Middle and right panel

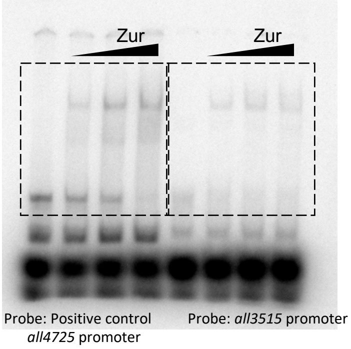

Image captured with a Cyclone Plus Phosphor System (Perkin Elmer)

Fig 2B top panel

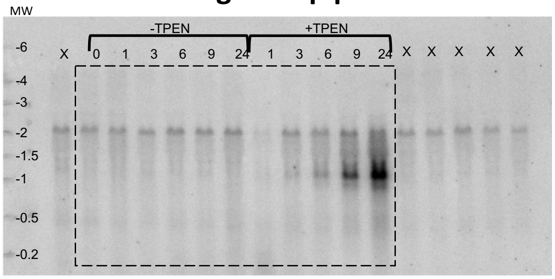

Image captured with a Cyclone Plus Phosphor System (Perkin Elmer)

Fig 2C top panel

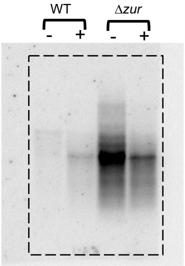

Image captured with a Cyclone Plus Phosphor System (Perkin Elmer)

Fig 2B bottom panel

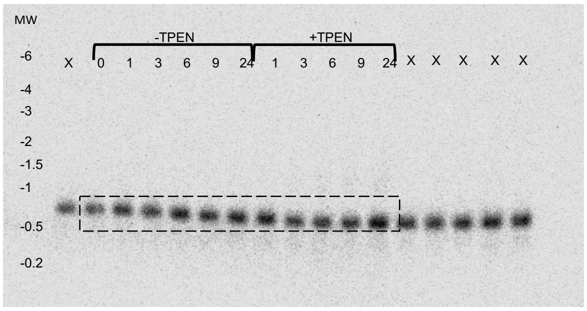

Image captured with a Cyclone Plus Phosphor System (Perkin Elmer)

Fig 2C bottom panel

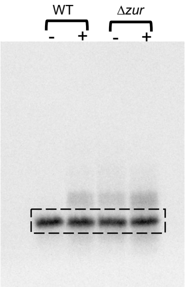

Image captured with a Cyclone Plus Phosphor System (Perkin Elmer)

Fig 4B Top left panel

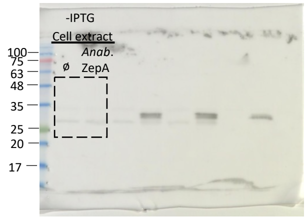

Image captured with an Amersham ImageQuant 800 equipment

Fig 4B Top right panel

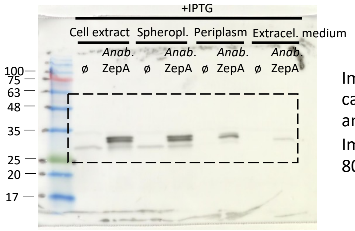

Image captured with an Amersham ImageQuant 800 equipment

Fig 4B Bottom left panel

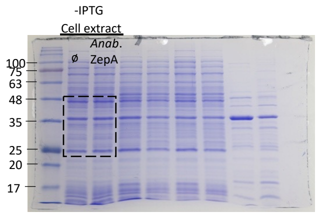

Image captured with a Canon EOS100D camera

Fig 4B Bottom right panel

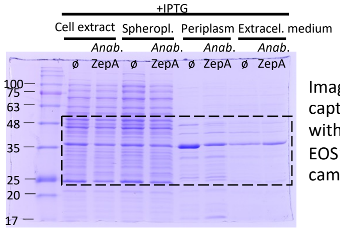

Image captured with a Canon EOS100D camera

Fig S2C

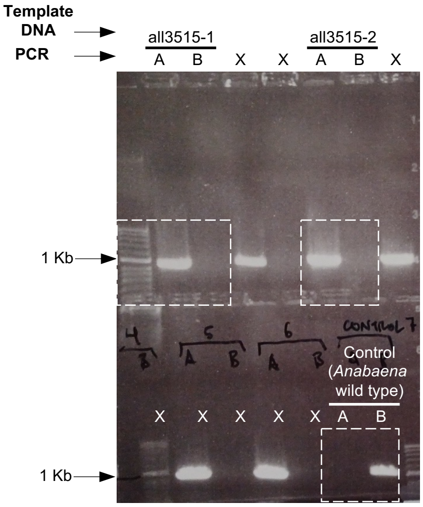

Image captured with GelDoc EZ Imager (Bio-Rad)

Fig S2D top, left lanes

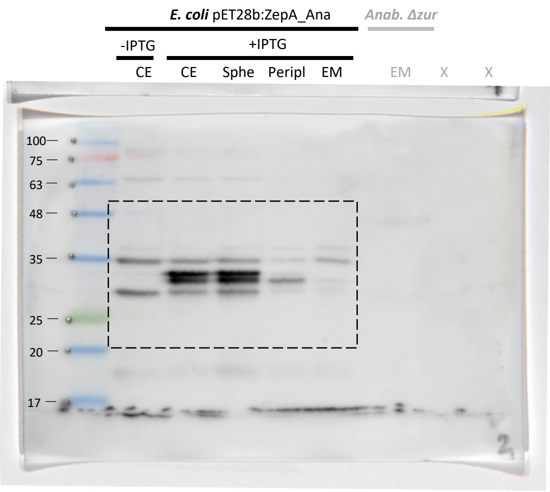

Fig S2D top, right lane

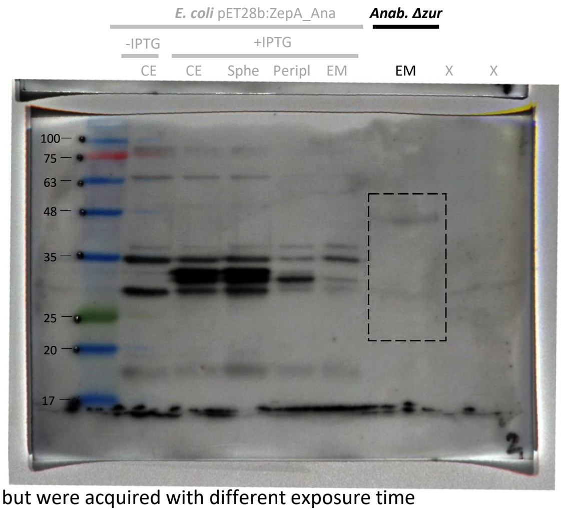

These two images correspond to the same blot, but were acquired with different exposure time

Images captured with an Amersham ImageQuant 800 equipment

Fig S2D bottom panel

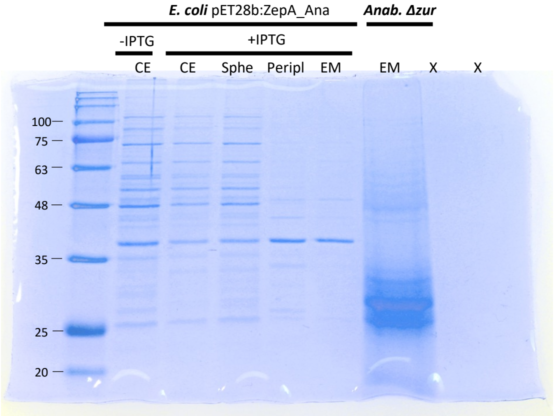

Image captured with a Canon EOS100D camera
