# Supplementary material for: An ancient bacterial zinc acquisition system identified from a cyanobacterial exoproteome
Source: PLoS Biol. 2024 Mar 11;22(3):e3002546. doi: 10.1371/journal.pbio.3002546 (PMC10957091; doi:10.1371/journal.pbio.3002546)
Supplement: Attachment — Submitted filename: Response to reviewers.docx [file pbio.3002546.s020.docx]

Reviewer #1:

The manuscript proposed by Sarasa-Buisan and co-authors is a good piece of research that characterizes the Zur-controlled exoproteome and its role in zinc acquisition by bacteria. Unfortunately, several shortcomings have been identified and clarifications are needed.

Major concerns:

1)	The authors should exploit the shotgun proteomics data in order to search for the exact coverage of the ZurA protein and establish the exact maturation sites (N-terminal signal peptide processing and C-terminal PEP-Cterm domain excision). Alternatively, they could analyze the global molecular weight of the proteins (LC-MS) and establish the exact limits of the ZurA polypeptide.

**We have thoroughly examined the proteomic data but only peptides in the central region of ZepA were identified, none of them proximal to the expected processing sites. In silico trypsin digestion of the ZepA sequence predicts a quite long N-terminal peptide (5776,91 Da) which makes difficult its detection. Similar situation occurs at the C-terminal region: in silico digestion predicts a very long peptide (5058,58 Da) along with multiple one and three residues short peptides (unlikely to be seen in the working m/z range of the instrument).**

**In this new version of the manuscript, we have developed specific antibodies against ZepA and we show that the exportation of this protein to the extracellular space is associated to processing by a double cleavage, which would be consistent with maturation of ZepA at both termini (see the new Fig. 4A, B). We agree that mapping of processing sites would be interesting, but we believe the main message of this manuscript is self sustained without this information, which could be sought in future work.**

2)	The authors did not demonstrate what is the quaternary structure of ZurA and whether ZurA binds zinc, and did not confirm the number of zinc atoms per protein. This would be an important result to complement their study, easily obtained after heterologous expression of the zurA gene.

**Evidence compatible with a monomeric conformation of recombinant ZepA is now provided, ICP-OES results indicating that ZepA specifically binds zinc are presented and the stoichiometry of zinc atoms per ZepA molecule was calculated. All this information is shown in the new Fig. 4C, D, E.**

3)	In the results section, the description of the proteomic experiment (lines 127-135) is not so clear. What was the efforts in terms of mass spectrometry? The number of MS/MS spectra recorded and the number of MS/MS spectra assigned to peptide sequences? How many biological replicates have been documented? What was the reproducibility of the analysis on these replicates? The authors mentioned in the M&M section the use of DDA and DIA analysis. The results section is unclear regarding these two strategies. Why were both strategies needed? Are the outcomes of the DDA and the DIA consistent?

**In the new version of the manuscript, we have clarified the description of the proteomic experiment as suggested, also indicating the number of biological and technical replicates used in the experiment (two and three, respectively). We have also included a new Figure (Fig. S1) showing the reproducibility of the quantitative proteomic data in both biological replicates. Also, we have now included the number of MS/MS spectra recorded and assigned to peptides in both DDA spectral library and DIA experiment. Finally, we explain in more detail that DDA data was used to build a spectral library to assist and improve DIA protein identification.**

4)	Here, why was the cellular proteome not analyzed at the same time? It would be interesting to highlight the proteins more specifically detected in the exoproteome compared to the cellular proteome with an enrichment factor. This would allow to highlight proteins that could come from cell lysis.

**We agree that a comparative analysis of the exoproteome vs. the cellular proteome is a wise strategy, particularly for the initial characterization of the exoproteome of an organism. While previous descriptions of the exoproteome of *Anabaena* are available (ref. 29, 30), the aim of our work was to understand the role of the exoproteome in the zinc acquisition process, a function poorly studied. With this aim, we performed a comparative analysis of the exoproteomes of two strains. Several lines of evidence make us confident that most proteins identified in this work do not derive from cell lysis: (i) the significant overlap of the proteins identified in our study with the exoproteomes previously published for *Anabaena*, (ii) the enrichment of the set of proteins identified in proteins with exportation signals and (iii) The absence of pigmentation in our preparations. Notice that cell lysis in cyanobacterial cultures provokes the liberation of the photosynthetic antenna complex that may account for 40-50% of the total protein content. These pigmented proteins are stable and easily detected by their intense blue color in the culture medium even if cell lysis occurs at minimal proportions. Our cultures were carefully inspected before proceeding to the preparation of samples for MS analyses. Besides, we only detected marginal contamination of a few of these proteins in the exoproteomes characterized in this work.**

**Also, in the new figure 1C included in this version we show that ZepA was detected in the culture medium but not in cell extracts, suggesting that its presence in the extracellular space is not due to cell lysis.**

5)	Figure 2 shows that the strategy for analysing vesicle protein content is different from the exoproteome analysis. The authors should have performed shotgun proteomics also on these vesicles in order to gain more insights. The rationale of this experimental change is difficult to understand.

**This part has been eliminated from the manuscript following a suggestion from reviewer 3**

Minor concerns

6)	The abstract needs rephrasing to improve its interest and be more exact. The abstract should gain clarity with the following rephrasing: "Bacteria have developed fine-tuned responses to cope with potential zinc limitation. The Zur protein is a key player in coordinating this response in most species. Comparative proteomics conducted on the cyanobacterium Anabaena highlighted the more abundant proteins in a zur mutant compared to the wild type. Experimental evidence showed that the exoprotein ZepA mediates zinc uptake. Genomic context of the zepA gene and protein structure prediction prvided additional insights. Phylogenetic analysis suggests that ZepA represents a primordial system for zinc acquisition that has been conserved for billions of years in a handful of species from distant bacterial lineages. Furthermore, these results show that Zur may have been one of the first regulators of the FUR family to evolve, consistently with the scarcity of zinc in the ecosystems of the Archean eon.".

**We are grateful to the reviewer for his/her effort in re-phrasing the abstract of our manuscript, which in this new version reads as he/she suggested.**

7)	In the introduction, the sentence "The external medium also contains proteins that constitute the so-called exoproteome" requires a general reference because the audience may not be familiar with the meaning of the exoproteome.

**Changed as suggested.**

8)	The sentence "Zur has been well characterized in a few species. Remarkably, in the oceanic cyanobacterium Synechococcus sp. WH8102, Zur exhibits unique features regarding the orientation of the two domains and the regulatory site, which is distinct and involves coordination residues different from those in non cyanobacterial Zur." needs also a reference.

**Changed as suggested.**

9)	Line 153: replace ORF by CDS if you are talking about polypeptide sequence.

**Changed as suggested.**

10)	Line 167: please indicate the fold change threshold.

**Changed as suggested.**

Reviewer #2:

Sarasa-Buisan et al. present data that identify exoproteome proteins that show changes in abundance, up or down, in a zur mutant strain. These data are used to identify a protein they have named ZepA that is involved in zinc handling. They show that ZepA is regulated by Zur and identify Zur binding sites close upstream of the TSS1 and TSS2. Growth experiments show that ZepA is required for growth when zinc is removed by chelation and protects against high zinc levels. Bioinformatics supports a ZepA relationship with zinc handling and phylogenetics indicated that ZepA may have evolved very early for zinc acquisition and that Zur may be one of the first members of the Fur family. This paper is thorough and presents a complete story; it is well organized and is well written. I found no major flaws in the experimental design, data, or presentation in the manuscript and have only a few minor comments for the authors to consider in revising their manuscript.

The following comments should be considered by the authors.

L 48. Replace "consistantly" with "consistant".

**In this new version we have replaced the abstract by a new one entirely rephrased by reviewer 1, who kept “consistently” in this sentence.**

L 255, Fig. 3C. The authors should comment on the effect of TPEN in the zur mutant. Maybe I missed it, but is there a hypothesis for the reduced level of transcripts in the presence of TPEN?

**TPEN is a non-specific chelator that permeates into the cell, provoking a systemic metal deficiency. Competition with RNA polymerase, a zinc-specific enzyme, is most likely the basis for some decrease in transcript levels. In previous work, we have observed that this effect is general for all transcripts and it is most evident at short time points. Despite this, TPEN does not abrogate transcription and does not impede the analysis of Zur-regulated gene expression.**

**This new version of the manuscript includes a comment on this issue.**

L 302. "Zur-dependent induction" is confusing because Zur is a repressor. This should be rephrased.

**“induction” has been replaced by “de-represion”**

L 357. The red arrows in Fig.6 are too small.

**Fixed**

L 372. "lane" should be "line".

**Fixed**

L 602. "obeyed to" is unclear here. This needs to be rephrased.

**Changed as suggested.**

L 664. The degree symbol is an "a" here.

**Fixed**

Supplementary Fig. S1 legend. Fix "estrategy"

**Changed as suggested.**

Supplementary Fig. S2 legend. Fix "confronted"; and "seldom" should probably be "rare".

**Done**

Reviewer #3:

In this manuscript the authors describe a study which uses an exoproteomic profiling comparing wild type Anabaena and a zur mutant to characterize extracellular zinc response. They also identified proteins in extracellular vesicles. They find a novel exoprotein overexpressed in the mutant, which they functionally characterize with EMSA to show Zur binding, Northern blots to show overexpression in the Zur mutant, and knockout mutant phenotyping. They then conduct bioinformatic analyses to find homologs of the new protein, genomic context of the homologs, phylogenetic analyses, and structural modeling. They conclude that the new protein ZepA mediates Zn uptake and interacts with the Zur regulator and that it is an ancient protein that was present in the LCBA and was lost in most lineages.

This manuscript represents an impressive amount of work and has some interesting and valuable results in characterizing a new putative Zn homeostasis protein. However I have some major concerns:

1.	It currently feels like 3-4 different stories (exoproteome, extracellular vesicles, ZepA function in Anabaena, ZepA evolution), based on only a few actual experiments which feel somewhat incomplete and/or incompletely described. I think the experimental results most clearly suggest a focus on the functional role of ZepA in Anabaena—all the other pieces are distracting and periphery, with much less support from the data. This is most clear in the Discussion, which needs to be narrowed and focused. E.g. the results presented do not justify an entire page on how exo-proteins reach the extracellular space, since the experiments were not designed to test this—there was 1 experiment on exoproteins that did not compare to intracellular proteomes and only ran duplicate biological replicates at one time point under one condition. The experiment compared the wild type and zur mutant so that is the data that should be the focus.

**The present version of the manuscript provides new experimental data strengthening the functional characterization of ZepA respect to the previous version and we have re-written significant portions of the Discussion to focus on the actual results of the manuscript.**

2.	The experimental results are intriguing, but do not go far enough to fully explain and characterize ZepA function—ZepA is supposedly extracellular, and thus you would expect a very different phenotype on solid vs liquid media, but the limited phenotyping of the mutant was done on solid medium, and not quantified statistically for significance. This feels like a missed opportunity, which ideally could be rectified, but at the very least should be noted. Further, a schematic model showing the proposed mode of action/regulation of ZepA in Anabaena Zn homeostasis should be added to aid interpretation of the results and what the unknowns still are. And possibly also compared to Zn homeostasis in another organism that has lost ZepA.

**In this version of the manuscript new experimental data that support that ZepA is an extracellular protein (Fig. 1C and 4B) that binds zinc in the extracellular space (Fig 4E and Fig. 8) are provided.**

**Phenotypic characterization of the mutants on solid media was repeated three times with similar results, which is now indicated in the manuscript.**

**A scheme on the proposed mode of action/regulation of ZepA in *Anabaena* is now included, clearly indicating the aspects that require further investigation.**

3.	ZepA evolution—this feels like a tangent, and while interesting, is not supported by the experimental results presented. The experimental work is done in one strain, and then the bioinformatic work extrapolates the same function to all the other homologs (e.g. line 575 "the apparent conservation of the ZepA function")—I think this needs to be better constrained in the interpretation since the function of the other homologs was not tested experimentally, nor are there experimental results in the literature to support the function of ZepA in the other taxa. Yes, the genomic context and structural modeling suggests the ZepA homologs also play a role in Zn homeostasis, this is still just a hypothesis until there is some experimental results. To then take the known homologs and discuss the evolution of this novel protein throughout the bacterial tree of life is too far without characterizing at least one other member.

**Genomic context and structural modeling but also the high sequence identity, the conservation of N- and C-terminal exportation signals, the conservation of zinc-binding residues and the presence of putative ZBSs upstream of *zepA* genes indicate that ZepA homologs are actual orthologs.**

**In an effort to provide experimental evidence on this issue, in this new version of the manuscript we present results showing that ZepA and ZepA homologs act similarly in the extracellular space (new Fig. 8). We want to stress that our first approach was to complement the *Anabaena* *all3515* (*zepA*) mutant with *zepA* genes from other species. Unfortunately, this was precluded by the apparent toxicity associated to minute expression of these genes in *E. coli* (notice that transfer of DNA into *Anabaena* by conjugation requires passage through 2 distinct *E. coli* strains). However, an assay was developed to test the functionality of ZepA homologs using a tightly controlled expression system and an *E. coli* strain that has revealed tolerant to the expression of ZepA. The assay was based on the observation that expression of *Anabaena* ZepA in this host provoked lethality in minimal medium in the presence of EDTA, which could be suppressed by the addition of excess zinc. ZepA accumulated in the culture medium, suggesting that zinc was captured by ZepA in the extracellular space, but the likely absence of downstream partners in this host impeded zinc reaching the cytoplasm, causing lethality. ZepA homologs from other bacterial lineages behaved in this assay similarly as *Anabaena* ZepA, evidencing the specificity of these ZepA homologs for zinc and their capacity to sequester zinc cations in the extracellular medium.**

More detailed comments:

Exoproteomes:

1.	Need to address cell lysis as a possible origin for the exoproteins, especially given the culturing to late exponential. Best would be to compare to intracellular proteomes, although this might not exist for the zur mutant.

**The intracellular proteome for the *zur* mutant is not available. However, we are confident that the impact of cell lysis on our exoproteome preparations is minimal since in cyanobacterial cultures cell lysis provokes the liberation of the photosynthetic antenna complex that may account for 40-50% of the total protein content. These pigmented proteins are easily detected by their intense blue color in the culture medium even if cell lysis occurs at minimal proportions. Our cultures were carefully inspected before proceeding to the preparation of samples for MS analyses. Besides, we only detected marginal contamination of a few of these proteins in the exoproteomes characterized in this work. See also response to point 4 by reviewer 1**

2.	Also need to be explicit that it is duplicate biological replicates (triplicates are usually required for statistical analyses), but at the very least make it clear in figure legends and results, and I think some of the "quantitative comparison" statements should probably be re-phrased.

**This is now clearly indicated in the text and statements on quantitative comparisons have been rephrased.**

3.	I also have some concern that the mutant may have different cell lysis rates than the wild type, so you could expect more extracellular proteins in the mutant than the wild type, or that if the mutant and wild type grew differently and were only sampled at one time point then you would see more intracellular proteins in one strain vs the other—a time course could have helped with this, but at the very least provide details on Zn levels and growth rates of both strains for that experiment.

**We agree that differential cell lysis rates may bias the comparative analysis of the exoproteome of both strains but several** **lines of evidence argue against this possibility: (i) we did not observe pigment accumulation in the extracellular medium of any of the two strains, (ii) the exoproteome of both strains was enriched in proteins with secretion or exportation signals and (iii) we have developed antibodies against ZepA and in this new version we present western blot results showing that ZepA is detected in the culture milieu but not in cell extracts indicating that its presence in the extracellular space is not due to cell lysis (Fig. 1C). Moreover, if the ∆*zur* mutant were more prone to cell lysis, we would expect an enrichment of intracellular proteins (about 3000 proteins in *Anabaena*) in the extracellular space, however we only detect six proteins significantly over-represented in the mutant exoproteome.**

**Zinc levels were 0.77 micromolar in all cultures throughout the experiment. Growth rates of both strains were monitored in this experiment and they were similar, as we have reported previously when these strains are cultured in standard conditions (see reference 25).**

4.	I'm not sure I'm convinced that ZepA only functions extracellularly—since they didn't compare to intracellular proteomes, it could be more abundant inside the cells—at the very least they could look in the literature for proteomics to see if it is detected intracellularly as well?

**As indicated above, new experimental results show that ZepA is only detected in the extracellular milieu. Other observations argue against ZepA being functional in the cytoplasm. For instance, the presence of a N-terminal signal peptide for the Sec pathway indicates that this protein would be exported before folding, so that ZepA would exist in the cytoplasm only transiently as an unfolded inmature precursor (Fig. 1C).**

5.	All the information on the exoproteins aside from ZepA are not presented in a clear fashion, all the TSS start site data (Lines 166-191) is very difficult to read through. Some more concise description would help, integrating with the next sections on ZepA.

**This part has been condensed and rephrased for the sake of clarity.**

6.	Methods:

a.	why continuous light?

**We used standard conditions for culture, as most other cyanobacterial labs do. Notice that culture under alternating light/dark cycles imposes a rhythmicity to cyanobacterial physiology that may add complexity to the analysis of particular phenomena.**

b.	How did the authors ensure late exponential growth and not stationary?

**We monitor cultures growth in all our experiments by meassuring the OD750 and the chlorophyll content. Samples used for exoproteome characterization in this manuscript were collected at OD750=1-1.3 (~8 µg chlorophyll ml^-1^), still far form the saturation of the growth curve (OD750>2 under standard growth conditions). Previous reports on *Anabaena* exoproteome were also performed using samples from late exponential phase cultures (see e.g. references 29 and 30).**

c.	What was the Zn concentration in the media in the exoproteome experiment?

**The concentration of zinc in the BG11 medium is 0.77 micromolar**

d.	Why dialyzed and then precipitated? Was this similar to the protocol compared to in the other papers cited?

**Dialysis of the lyophilized-concentrated exoproteome samples was performed to get rid of insoluble salts from culture media that remained even after protein digestion and precipitation by the proteomics facility. This insoluble material provoked significant overpressure issues in the chromatography apparatus, making impossible the processing of the sample. A parallel approach using preparative gel filtration PD-10 columns also proved effective to solve this problem, however the dialysis approach yielded overall superior performance in protein identification. By contrast, the use of membrane concentrators as an alternative approach provoked a mucous biogel-like consistency and was rejected.**

  Extracellular Vesicles:

This part is not integrated well at all with the rest of the paper, and not completely described in the methods or results—I would suggest removing it entirely. The method for extraction appears to be general for bacteria (although it was not detailed at all and merely references the paper)—was there any verification of whether it was successful or biased? As I understand vesicle extraction is not straightforward between different taxa and extraction may need to be modified.

**Following the suggestion of the reviewer, this part has been removed**

ZepA function:

EMSA: Isn't there usually a negative control too, not just a positive?

**We included a negative control in our experiment and it is now shown in Fig. 2A**

For the RNA and Northern extracts, methods are sparse—one publication referenced, and not in that organism. Also no information on replicates or any brief description of the methods.

**This is now thorougly described in Materials and Methods and the number of replicates is indicated in the figure legend.**

No description of the methods for phenotyping the mutants—were replicates run? That data is not quantitative thus difficult to know whether interpretation is correct. No statistics on that analysis either.

**Details on these experiments are now included in the Materials and Methods section and the number of replicates (n=3) is now indicated in the figure legend.**

Genome context, phylogeny and structural modeling:

1.	The genome context work is nice and does support and similar role for the homologs (although this still needs to be verified experimentally)

**As explained above, in this new version of the manuscript we provide experimental evidence supporting that *Anabaena* ZepA and ZepA homologs from other lineages are specific for zinc and bind this metal in the extracellular space. Notice that technical reasons impeded other experimental approaches (i.e.complementation of *zepA*/*all3515* mutants of *Anabaena*).**

2.	The phylogeny part is outside of my expertise, but with so few homologs the interpretation that the 16S and gene trees are similar does not seem that compelling to mean that vertical transmission is likely, we may just be missing key taxa.

**The reviewer suggests increasing the phyla number or sequence diversity for robustness, but the sparse distribution of *zepA* across phyla limited our ability to do so. This constraint notwithstanding, our analyses are sufficiently robust to provide significant insights into the *zepA* gene's evolutionary trajectory. Our analyses are based on all available *zepA* homologs (48 sequences) retrieved from the Refseq select NCBI database. While fine details of the evolutionary trajectory of a gene may be blurred due to the reduced number of species considered, large evolutionary anomalies as interphyla HGT would be readily detected. However, our analyses indicated that such phenomenon is rare in the evolution of *zepA*, which may be related with its observed toxicity in a heterologoust host. Furthermore, our assertion that the *zepA* gene tree is not significantly different from the species tree, thereby supporting the hypothesis of predominant vertical inheritance, is grounded in a comparative analysis of the confidence sets derived from each phylogenetic reconstruction. While phylogenetic reconstructions typically emphasize the most likely tree, or the primary topology, they also yield a range of plausible alternative trees, known as a confidence set. This set encompasses various potential topologies. In our analysis, we determined that the gene tree topology aligns with the species tree if the species tree's topology falls within the confidence set generated from the gene tree reconstruction. This overlap indicates that the differences between the gene tree and the species tree topologies are not significant (Fig. S9), reinforcing the likelihood of vertical inheritance patterns in the *zepA* gene. As detailed in the Materials and Methods section, “we used AU-test as implemented in W-IQ-TREE to estimate the P-value of the species tree topology to be in the confidence set of *zepA* trees.”**

3.	The structural modeling is also nice, as is the prediction of metal binding, and provides some good hypotheses to test experimentally. The figures and text could both be made more concise.

**In this new version of the manuscript we have made an effort to be more concise.**

4.	All of these pieces could be made more concise and constrained better to the experimental results and literature to make a more clear story.

**In this new version of the manuscript we have made an effort to be more concise and more focused on the experimental results.**
