# Supplementary material for: An ancient bacterial zinc acquisition system identified from a cyanobacterial exoproteome
Source: PLoS Biol. 2024 Mar 11;22(3):e3002546. doi: 10.1371/journal.pbio.3002546 (PMC10957091; doi:10.1371/journal.pbio.3002546)
Supplement: Attachment — Submitted filename: Response to reviewers.docx [file pbio.3002546.s021.docx]

**We would like to thank the reviewer for insightful comments that have very much helped to improve our manuscript.**

Reviewer #3:
[identifies herself as Rhona K. Stuart]

The manuscript is much improved, and most of my concerns have been addressed. 

One that I don't think was fully addressed is regarding the phenotyping shown in Figure 3—where is the data for the other times it was run? It's not enough to just say it was replicated three times. At the very least put it all in supplemental so that the reader doesn't have to take it on faith. But even better, instead of pictures all that data could be averaged and quantified and some stats tests for significance applied.

**We have assessed the growth of the three strains by quantifying the cell density in the spots on the plates used in the three replicates of the experiments in Fig. 3. We have defined and calculated a Growth Index (GI), which is indicative of the growth capacity of each strain in a particular medium. The average GI was represented in the heatmaps of the new Fig. S10 and all numerical data and calculations are provided in the new file S2_Data.** **Statistical analysis was performed to deduce which changes were significant and is included in Fig. S10. The procedure followed for quantification and calculation is described in a new section in Materials and Methods (lines 743-754).**

Also, I made a point in my previous review about the solid vs liquid media comparisons, and still think there should be some mention of this in results/discussion—the discussion talks about structured environments retaining ZepA—Anabaena certainly expresses proteins and exoproteins differently in liquid versus plate growth, so these are not really equivalent.  It's interesting that you see a phenotype you expect in solid medium, but it should be noted in result or discussion this is a different cultivation environment than the exoproteomics experiment.

**In our study we were lucky to identify ZepA in liquid culture because of the use of a ∆*zur* strain. In this non-natural strain, the absence of Zur provokes constitutive expression and secretion of ZepA, whichever the medium (solid or liquid) or condition used. By contrast, in a wild type strain, ZepA would be only expressed under zinc limitation conditions. In most natural environments zinc is apparently not limiting, but zinc deficiency would be encountered in habitats where competition for nutrients is strong, including structured environments like microbial mats. Therefore, in nature ZepA would be expressed and secreted precisely in those environments where this protein is expected to be more effective for zinc acquisition because its diffusion is limited. This issue is discussed in this new version of the manuscript in lines 628-634.**

I think the ZepA evolution has been better described and qualified.  I think it would be even more convincing to look at genomes from metagenomics datasets, or just look for ZepA occurrence in those datasets. For the hypothesis that only "structured environments" would retain ZepA, you wouldn't expect to see it in aquatic datasets but yes in mats or soils. However, this is more of a suggestion, (which would help convince me of what the authors argue regarding it's evolution).

**We appreciate the suggestion to investigate the distribution of *zepA* in current metagenomes. However, this topic deserves more than just a mention in the article; it requires a comprehensive study that integrates metatranscriptomes. We plan to address this point in a more detailed manner in the near future.**

Minor comments:
Line 46:  "additional insights" is very vague.  Consider specifying.

**We have completed the sentence that now reads “Genomic context of the *zepA* gene and protein structure prediction provided additional insights on the regulation and putative function of ZepA homologs”**

Line 49: "Consistently" to "consistent".

**Changed as suggested**

Line 86: "zinc traces" to "trace zinc"

**Changed as suggested**
